# STAT3 inhibition suppresses adaptive survival of *ALK*-rearranged lung cancer cells through transcriptional modulation of apoptosis

**DOI:** 10.1038/s41698-022-00254-y

**Published:** 2022-02-28

**Authors:** Naohiro Yanagimura, Shinji Takeuchi, Koji Fukuda, Sachiko Arai, Azusa Tanimoto, Akihiro Nishiyama, Naohisa Ogo, Hiroyuki Takahashi, Akira Asai, Satoshi Watanabe, Toshiaki Kikuchi, Seiji Yano

**Affiliations:** 1 Division of Medical Oncology Cancer Research Institute, Kanazawa University, Kanazawa, Japan; 2 Department of Respiratory Medicine and Infectious Diseases, Niigata University Graduate School of Medical and Dental Sciences, Niigata, Japan; 3 Nano Life Science Institute, Kanazawa University, Kanazawa, Japan; 4 Center for Drug Discovery, Graduate School of Pharmaceutical Sciences, University of Shizuoka, Shizuoka, Japan; 5 Pharmaceutical Business Division, Yakult Honsha Co. Ltd, Tokyo, Japan

**Keywords:** Non-small-cell lung cancer, Targeted therapies

## Abstract

Patients with advanced anaplastic lymphoma kinase (*ALK*)-rearranged non-small cell lung cancer who are prescribed ALK-tyrosine kinase inhibitors (ALK-TKIs) rarely have complete responses, with residual tumors relapsing as heterogeneous resistant phenotypes. Herein, we investigated new therapeutic strategies to reduce and eliminate residual tumors in the early treatment phase. Functional genomic screening using small guide RNA libraries showed that treatment-induced adaptive survival of *ALK*-rearranged lung cancer cells was predominantly dependent on STAT3 activity upon ALK inhibition. STAT3 inhibition effectively suppressed the adaptive survival of *ALK*-rearranged lung cancer cells by enhancing ALK inhibition-induced apoptosis. The combined effects were characterized by treatment-induced STAT3 dependence and transcriptional regulation of anti-apoptotic factor BCL-X_L_. In xenograft study, the combination of YHO-1701 (STAT3 inhibitor) and alectinib significantly suppressed tumor regrowth after treatment cessation with near tumor remission compared with alectinib alone. Hence, this study provides new insights into combined therapeutic strategies for patients with *ALK*-rearranged lung cancer.

## Introduction

Advances in molecular targeted therapies have dramatically improved the prognosis of patients with advanced non-small cell lung cancer (NSCLC) harboring driver oncogenes^1,2^. Echinoderm microtubule-associated protein-like 4 (*EML4*)-anaplastic lymphoma kinase (*ALK*) fusion is observed in 3–7% of patients with lung adenocarcinoma^3,4^. Since its discovery, several ALK-tyrosine kinase inhibitors (ALK-TKIs) have been developed and improved the clinical outcomes of patients with advanced *ALK*-rearranged lung cancer^5^. Specifically, second-generation ALK-TKIs (e.g., alectinib and brigatinib), which have demonstrated more promising clinical efficacy than the first-generation ALK-TKI crizotinib in recent phase III clinical trials (J-ALEX, ALEX, and ALTA-1L), are currently used as first-line therapy^6–8^. Additionally, the third-generation ALK-TKI lorlatinib, approved for patients with a history of failed ALK-TKI treatments, has shown more promising clinical efficacy than crizotinib in a recent phase III clinical trial (CROWN)^9^. Despite the promising clinical efficacies of the new-generation ALK-TKIs, nearly all tumors relapse due to acquisition of resistance within a few years.

To date, multiple molecular mechanisms underlying acquired resistance to ALK-TKIs have been identified. For instance, secondary mutations in the ALK kinase domain (e.g., G1202R, V1180L, I1171T, and F1174C) have been detected in approximately half of all patients treated with second-generation ALK-TKIs^10–12^. Sequential acquisition of compound mutations (e.g., C1156Y/L1198F, G1202R/L1196M) is responsible for disease progression in approximately one-third of lorlatinib-treated patients^13,14^. Additionally, various *ALK-*independent resistance mechanisms have been reported in preclinical studies, including activation of bypass signaling and epithelial-to-mesenchymal transition^15–20^. Often tumors acquire resistance through multiple molecular mechanisms simultaneously, causing an increase in intra-tumor resistance heterogeneity in the later phase of therapy^10,12,13^. Therefore, elimination of tumors during the early phase of therapy before acquiring heterogeneous resistance is imperative. However, studies have demonstrated the difficulty in eliminating all tumors with single-agent ALK-TKI during the early phase of therapy; complete response has been observed in only a few cases (<5%), even with alectinib, the mainstay of first-line therapy considering its promising clinical efficacy high objective response rate (82.9–92%)^6,7^.

The mode of escape employed by residual cancer cells to avoid initial apoptosis and adapt to molecular targeted agents has recently been highlighted among researchers^21,22^. For instance, in epidermal growth factor receptor (*EGFR)*-mutant lung cancer, activation of the Hippo pathway effector protein YAP, Notch-3/β-catenin signaling, AXL, insulin-like growth factor 1 receptor (IGF-1R), or *BIM* deletion polymorphism mediates drug persistence to initial EGFR-TKI treatment^23–28^. However, in *ALK*-rearranged lung cancer, the molecular mechanisms underlying residual tumors remain largely unexplored.

In this study, we investigate the role of signal transducer and activator of transcription 3 (STAT3) in treatment-induced adaptive survival of *ALK*-rearranged lung cancer cells and evaluate the preclinical antitumor activities of STAT3-targeting combination therapy using *ALK*-rearranged lung cancer cell lines and a xenograft model. Our findings provide new insights into combined therapeutic strategies aimed at tumor eradication in *ALK*-rearranged lung cancer.

## Results

### Treatment-induced adaptive cancer cell populations survive upon ALK inhibition

**Fig. 1:**
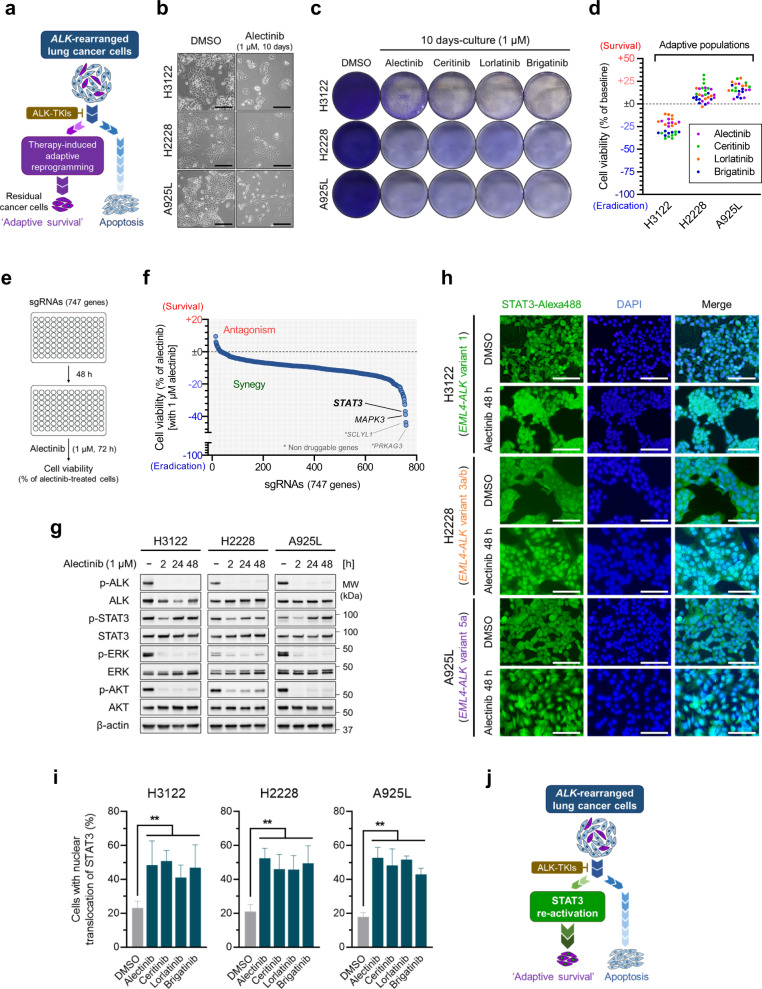
Treatment-induced adaptive survival of anaplastic lymphoma kinase (*ALK*)-rearranged lung cancer cells is dependent on STAT3 activity. **a** Schematic representation of the study design. **b** *ALK*-rearranged lung cancer cell lines, H3122 (*EML4-ALK* variant 1), H2228 (*EML4-ALK* variant 3a/b), and A925L (*EML4-ALK* variant 5a), treated with alectinib (1 µM) or dimethyl sulfoxide (DMSO) for 10 days. Images of control cells (at 5 days) or adaptive cells (at 10 days) are shown. Scale bars = 200 µm. **c** Adaptive populations of H3122, H2228, and A925L cells were visualized by crystal violet staining following 10-days incubation with ALK-TKIs (alectinib, ceritinib, lorlatinib and brigatinib: 1 µM). The drugs were replenished every 72 h. **d** Cell viability of the adaptive H3122, H2228, and A925L cells (*n* = 6–8, representing separate wells) was quantified by MTT assay. **e** Schematic representation of the functional genomic CRISPR-KO screening. **f** A925L/Cas9 cells were transduced with small guide RNAs (sgRNAs; targeting total 747 genes) for 48 h and treated with alectinib (1 µM) for an additional 72 h. Cell viability was quantified by MTT assay. The screening was performed once. **g** H3122, H2228, and A925L cells were treated with alectinib (1 µM) for the indicated hours and the lysates were analyzed by western blotting with the indicated antibodies. **h** Immunofluorescence staining with signal transducer and activator of transcription 3 (STAT3)-Alexa 488 and DAPI of H3122, H2228, and A925L cells treated with alectinib (1 µM) or DMSO for 48 h. Scale bars = 100 µm. **i** Quantification of STAT3 nuclear translocation in H3122, H2228, and A925L cells treated as in **h**. Significant differences were determined using one-way ANOVA with Dunnett’s multiple comparison tests (**p < 0.01). Data are shown as mean ± SD of experimental replicates; *n* = 4, representing different field. **j** Schematic representation of the hypothetical roles of STAT3 in the adaptive survival of *ALK*-rearranged lung cancer cells.

We designed in vitro experiments in this study to reduce and eliminate residual cancer cells maintained by treatment-induced adaptive reprogramming (Fig. 1a) using three *ALK*-rearranged lung cancer cell lines with different *EML4-ALK* variants (H3122, H2228, and A925L cells). The characteristics of each cell line, including *EML4-ALK* fusion protein expression and IC_50_ values of ALK-TKIs (alectinib, ceritinib, lorlatinib, and brigatinib) are shown in Supplementary Fig. 1. In all three cell lines, cell proliferation was suppressed at relatively low concentrations of ALK-TKIs (0.01–0.1 µM); however, higher concentrations of ALK-TKIs (0.3–3 µM) were required to reduce survival cells compared to the baseline (Supplementary Fig. 2a–d). Even after continuous incubation with 1 µM ALK-TKIs for 10 days, which is considered the clinically relevant concentration based on previously reported maximum plasma concentrations (*C*_max_: alectinib [300 mg BID], 1107.5 nM; ceritinib [750 mg QD], 1433.3 nM; lorlatinib [100 mg QD], 1400.1 nM; and brigatinib [90 mg QD], 945.1 nM; brigatinib [180 mg QD], 2485.4 nM)^29–32^, the viability of all three cell lines was retained >70% relative to the baseline (Fig. 1b–d). Thus, treatment-induced adaptive cancer cells were formed allowing for the maintenance of residual tumors upon initial ALK-TKI treatment.

### Adaptive survival of *ALK*-rearranged lung cancer cells is dependent on STAT3 activity

To explore the molecular mechanisms underlying treatment-induced adaptive survival of *ALK*-rearranged lung cancer cells, we performed functional genomic CRISPR KO screen using small guide RNA (sgRNA) libraries (Fig. 1e). Across the sgRNAs (targeting 747 genes), STAT3 and MAPK3, as the druggable targets, showed maximum synergistic effect on decreasing cell viability of A925L cells (Fig. 1f and Supplementary Table 1). Therefore, we performed kinetic analysis of ALK downstream signaling, including MAPK/ERK, PI3K/AKT, and STAT3 in H3122, H2228, and A925L cells treated with alectinib, showing that while the phosphorylation of ALK, ERK, and AKT was suppressed through 48 h, the phosphorylation of STAT3 was restored within 24 h (Fig. 1g). When treated with the other ALK-TKIs (ceritinib, lorlatinib, and brigatinib), restored activation of STAT3 was similarly observed in the absence of ALK signaling (Supplementary Fig. 3a).

Considering that inactive STAT3 exists as a monomer in the cytoplasm and phosphorylation of Tyr705 results in SH2-domain-mediated head-to-tail dimerization of STAT3 and its subsequent translocation to the nucleus as a transcription factor^33^, we evaluated the intracellular dynamics of STAT3 upon ALK inhibition by immunofluorescence staining. In control cells, STAT3 was evenly distributed throughout the cytoplasm and nucleus, whereas following treatment with ALK-TKIs (alectinib, ceritinib, lorlatinib, and brigatinib), STAT3 more localized in the nucleus of H3122, H2228, and A925L cells (Fig. 1h, i and Supplementary Fig. 3b). Overall, our results indicate that the treatment-induced adaptive survival of *ALK*-rearranged lung cancer cells is predominantly dependent on STAT3 activity (Fig. 1j).

### STAT3 is required for survival of *ALK*-rearranged lung cancer cells upon ALK inhibition

**Fig. 2:**
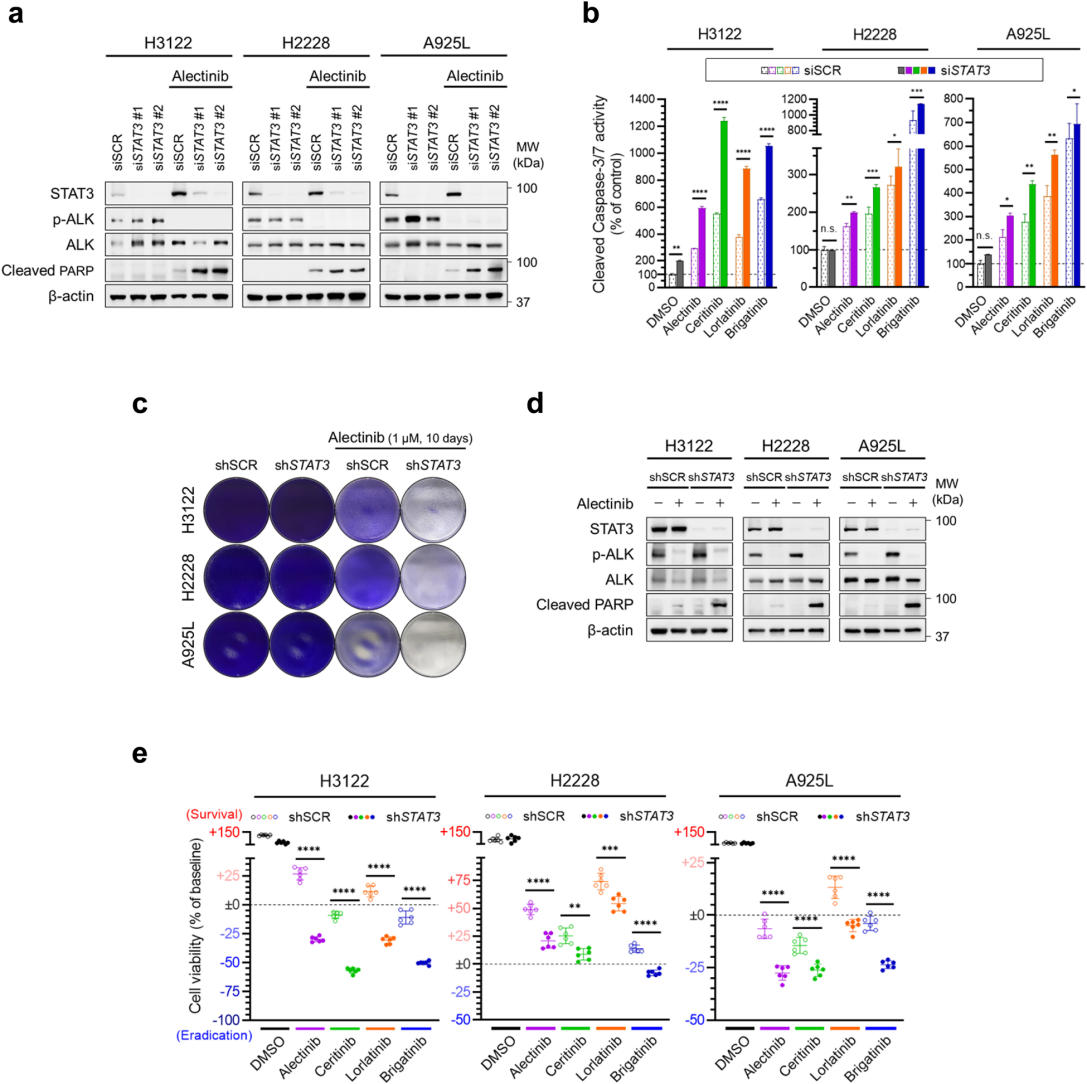
STAT3 is required for the survival of *ALK*-rearranged lung cancer cells upon ALK inhibition. **a** H3122, H2228, and A925L cells were transfected with siRNA targeting *STAT3* (si*STAT3*) or scramble siRNA (siSCR) for 48 h and treated with alectinib (1 µM) or DMSO for an additional 48 h. The lysates were analyzed by western blotting with the indicated antibodies. **b** H3122, H2228, and A925L cells were transfected with si*STAT3* or siSCR for 48 h and treated with ALK-TKIs (alectinib, ceritinib, lorlatinib, and brigatinib: 1 µM) or DMSO for an additional 72 h. Apoptosis was quantified using Caspase-Glo® 3/7 Assay. **c** H3122, H2228, and A925L cells transduced with shRNA targeting *STAT3* (sh*STAT3*) or non-targeting control shRNA (shSCR) were treated with alectinib (1 µM) or DMSO for 10 days. Viable cell populations were visualized by crystal violet staining. The drugs were replenished every 72 h. **d** H3122, H2228, and A925L cells transduced with sh*STAT3* or shSCR were treated with alectinib (1 µM) or DMSO for 48 h. The lysates were analyzed by western blotting with the indicated antibodies. **e** H3122, H2228, and A925L cells transduced with shSTAT3 or shSCR were treated with ALK-TKIs (alectinib, ceritinib, lorlatinib, and brigatinib: 1 µM) or DMSO for 72 h. Cell viability was quantified by MTT assay. Significant differences were analyzed using one-way ANOVA with Sidak’s multiple comparison tests; **p < 0.01, ***p < 0.005, and ****p < 0.001. Data are shown as mean ± SD of experimental replicates; *n* = 3 in **b** and *n* = 6 in **e**, representing separate wells. In all experiments, three independent experiments were performed, and representative results are shown.

We investigated the functional roles of STAT3 in the adaptive survival of *ALK*-rearranged lung cancer cells by evaluating apoptosis induction with *STAT3* knockdown. Transient *STAT3* knockdown by siRNA (si*STAT3*) suppressed alectinib-induced STAT3 re-activation and markedly induced apoptosis in H3122, H2228, and A925L cells (Fig. 2a). When treated with the other ALK-TKIs (ceritinib, lorlatinib, and brigatinib), si*STAT3* induced comparable increase in the expression of apoptosis markers, including cleaved caspase-3 and cleaved PARP (Supplementary Fig. 4a), as well as cleaved caspase-3/7 activity (Fig. 2b). We further examined the role of STAT3 in the survival of adaptive cells under long-term exposure to ALK-TKIs using shRNA to achieve stable *STAT3* knockdown (sh*STAT3*). After 10 days-culture in presence of ALK-TKIs, the residual cancer cells were markedly reduced with nearly eliminated by sh*STAT3* (Fig. 2c and Supplementary Fig. 4b). Western blotting confirmed stable *STAT3* knockdown and apoptosis induction (Fig. 2d). The survival cell numbers were significantly reduced by sh*STAT3* in all three cell lines treated with ALK-TKIs (Fig. 2e). Therefore, STAT3 mediates adaptive survival of *ALK*-rearranged lung cancer cells through evasion of apoptosis.

### A STAT3 inhibitor YHO-1701 suppresses the adaptive survival of *ALK*-rearranged lung cancer cells

**Fig. 3:**
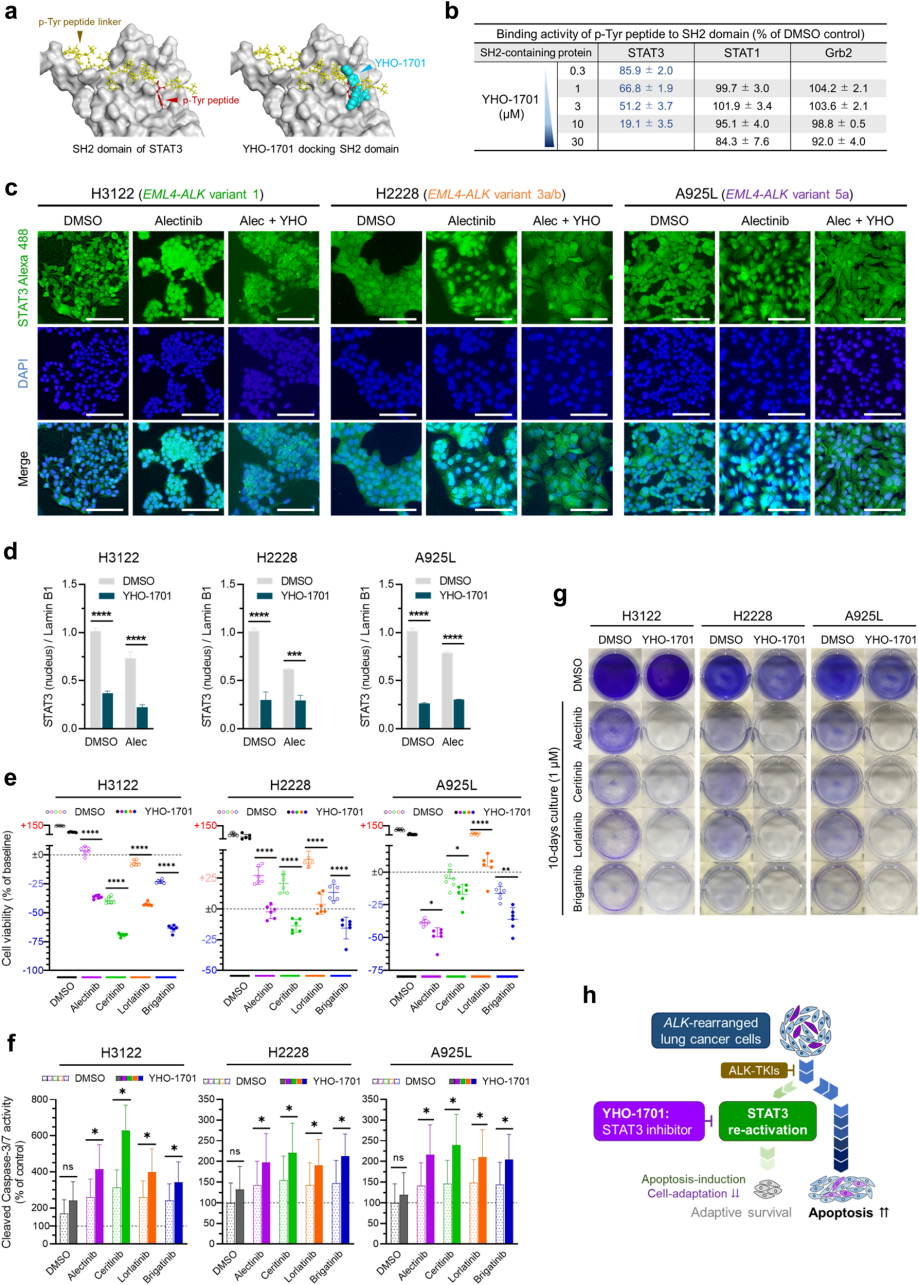
STAT3 inhibitor suppresses the adaptive survival of *ALK*-rearranged lung cancer cells. **a** Docking model: YHO-1701 in the Src homology 2 (SH2) domain of STAT3. Left: structural models of the SH2 domain of STAT3 with phospho-Tyr (pY) peptide linkers. The pY peptide linker for STAT3 is shown in yellow and pY as red in the ball-stick representation. Right: overlay of the pY peptide linker and YHO-1701 in the SH2 domain of STAT3 generated using MOE. YHO-1701 highlighted in cyan is shown in the space-filling representation. **b** Inhibitory activities of YHO-1701 against SH2-containing proteins. Binding activity of the phospho-Tyr peptide to the SH2 domain is shown as a percentage of DMSO control. **c** Immunofluorescence staining with STAT3-Alexa 488 and DAPI of H3122, H2228, and A925L cells treated with alectinib (1 µM) or DMSO with or without YHO-1701 (3 µM) for 48 h. Scale bar = 100 µm. **d** H3122, H2228, and A925L cells were treated as in **c**, and intra-nuclear expression of STAT3 were quantified relative to Lamin B1 using western blotting for nuclear fractions of the lysates in Supplementary Fig. 5C. **e** H3122, H2228, and A925L cells were treated with ALK-TKIs (alectinib, ceritinib, lorlatinib, and brigatinib: 1 µM) or DMSO with or without YHO-1701 (3 µM) for 72 h. Cell viability was quantified by MTT assay. **f** Apoptosis of the cells treated as in **d** was quantified using Caspase-Glo® 3/7 Assay. **g** Viable populations of H3122, H2228, and A925L cells were visualized by crystal violet staining following 10 days incubation with ALK-TKIs (alectinib, ceritinib, lorlatinib and brigatinib: 1 µM) or DMSO with or without YHO-1701 (3 µM). The drugs were replenished every 72 h. **h** Schematic representation of the results wherein a new STAT3 inhibitor, YHO-1701, suppresses the adaptive survival of *ALK*-rearranged lung cancer cells. Significant differences were analyzed using one-way ANOVA with Sidak’s multiple comparison tests in **d**, **e** and two-tailed Student’s *t*-test in **f**; *p < 0.05, **p < 0.01, ***p < 0.005, and ****p < 0.001. Data are presented as mean ± SD of experimental replicates; *n* = 3 in **b**, **d**, **f** and 6 in **e**, representing separate wells. In all experiments, three independent experiments were performed, and representative results are shown.

YHO-1701, a new STAT3 dimerization inhibitor, prevents the binding of phospho-Tyr peptide to the SH2 domain of STAT3. The phospho-Tyr peptide linker, which plays a crucial role in the dimerization of STAT3, fits in the cavity on the SH2 domain surface allowing YHO-1701 to dock the phospho-Tyr peptide binding site utilizing hydrogen bonding and hydrophobic interactions^34^, effectively covering the STAT3 SH2 domain cavity and interfering with phospho-Tyr peptide binding (Fig. 3a). However, before in vitro application, we ensured that the primary concerns associated with employing a new compound, namely target-selectivity and possible toxicity to non-tumor tissues, were not significant factors in our assays: YHO-1701 effectively inhibited the binding of phospho-Tyr peptide to the SH2 domain of STAT3 in a concentration-dependent manner with IC_50_ values of 2.5 µM for STAT3 and >30 µM for other adapter proteins containing SH2 domains, such as STAT1 and Grb2, thereby demonstrating high selectivity for STAT3 (Fig. 3b and Supplementary Fig. 5a). The inhibition ratios of YHO-1701 for the various receptors, including ion channels and transporters, were all less than 50% (Supplementary Tables 2 and 3). Moreover, the viability of human lung embryonic fibroblast cells MRC-5 and IMR-90, representing non-tumorigenic cells, decreased by ~20%, following exposure to varying concentrations (1–10 µM) of YHO-1701 (Supplementary Fig. 5b).

Subsequently, we assessed the pharmacological effects of YHO-1701 in *ALK*-rearranged lung cancer cells via inhibition of STAT3 nuclear translocation. STAT3 nuclear translocation was significantly inhibited following YHO-1701 addition in H3122, H2228, and A925L cells (Fig. 3c, d and Supplementary Fig. 5c). Additionally, YHO-1701 significantly reduced the survival of these cell lines treated with ALK-TKIs (alectinib, ceritinib, lorlatinib, and brigatinib) (Fig. 3e). Activity of cleaved caspase-3/7 and expression of apoptosis marker (cleaved PARP and cleaved caspase-3) increased significantly in cells treated with ALK-TKIs and YHO-1701 (Fig. 3f and Supplementary Fig. 5d). Furthermore, after 10 days of culture in presence of ALK-TKIs and YHO-1701, H3122, H2228, and A925L cells were almost eliminated (Fig. 3g).

Collectively, these results indicate that YHO-1701 effectively suppresses cell adaptation to ALK-TKIs driven by STAT3 re-activation and induces apoptosis in residual *ALK*-rearranged lung cancer cells (Fig. 3h).

### STAT3 mediates the evasion of apoptosis by promoting anti-apoptotic BCL-X_L_ expression

**Fig. 4:**
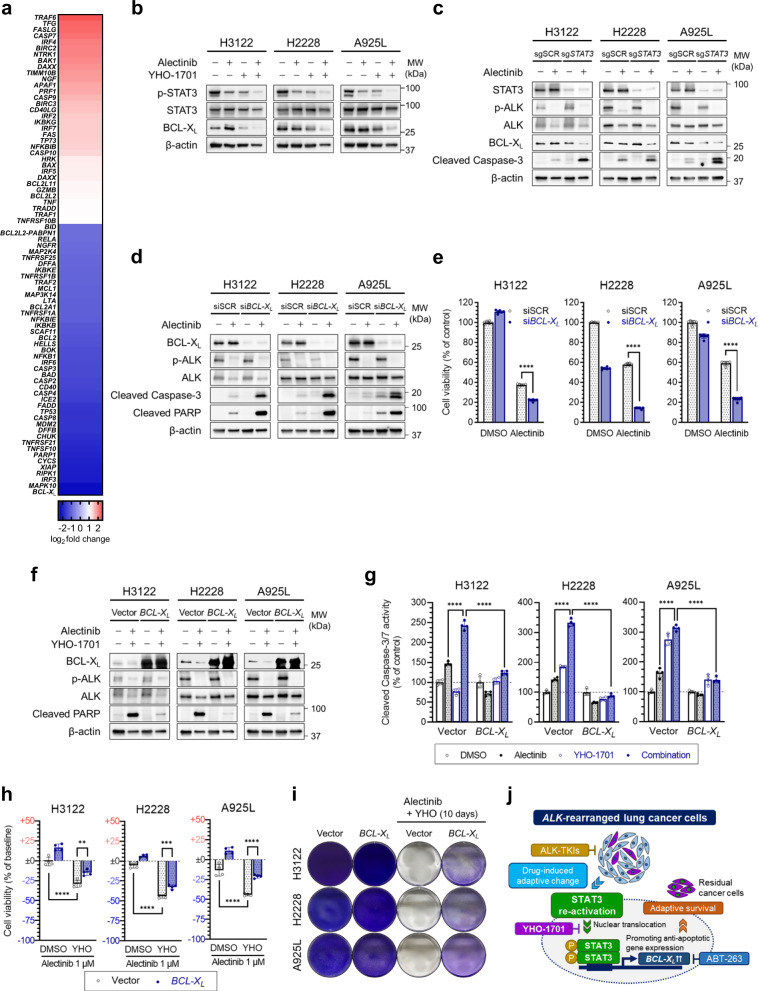
STAT3 mediates the evasion of apoptosis by promoting anti-apoptotic BCL-X_L_ expression. **a** Microarray analysis of normalized mRNA expression of apoptosis-related genes (total 78 genes) in H3122 cells treated with YHO-1701 (3 µM) for 24 h. Colors indicate log_2_ fold change values. The microarray analysis was performed once. **b** H3122, H2228, and A925L cells were treated with alectinib (1 µM) or DMSO with or without YHO-1701 (3 µM) for 48 h. The lysates were analyzed by western blotting with the indicated antibodies. **c** Non-targeting guide RNA-transduced (sgSCR) or *STAT3* knockout (KO; sg*STAT3*) H3122, H2228, and A925L cells were treated with alectinib (1 µM) or DMSO for 48 h. The lysates were analyzed by western blotting with the indicated antibodies. **d** Protein expression and **e** cell viability of H3122, H2228, and A925L cells transfected with siRNAs targeting *BCL2L1* (encoding the anti-apoptotic protein BCL-X_L_: si*BCL-X*_*L*_) or siSCR for 48 h and treated with alectinib (1 µM) or DMSO for an additional 48 h. The cell lysates were analyzed by western blotting with the indicated antibodies, and cell viability was quantified by MTT assay. **f** H3122, H2228, and A925L cells transduced with control or *BCL-X*_*L*_ overexpressing vector were treated with alectinib (1 µM) and YHO-1701 (3 µM) or DMSO for 48 h. The cell lysates were analyzed by western blotting with the indicated antibodies. **g** Apoptosis and **h** viability of H3122, H2228, and A925L cells transduced with control or *BCL-X*_*L*_ overexpressing vector and treated with alectinib (1 µM) or DMSO with or without YHO-1701 (3 µM) for 72 h. Apoptosis was quantified using Caspase-Glo® 3/7 Assay, and cell viability was quantified by MTT assay. **i** H3122, H2228 and, A925L cells transduced with control or *BCL-X*_*L*_ overexpressing vector were treated with alectinib (1 µM) and YHO-1701 (3 µM) or DMSO for 10 days. Viable cell populations were visualized by crystal violet staining. The drugs were replenished every 72 h. **j** Schematic representation of the results: STAT3 facilitates the evasion of apoptosis in anaplastic lymphoma kinase (*ALK*)-rearranged lung cancer cells upon ALK inhibition by inducing anti-apoptotic protein, BCL-X_L_, leading to “adaptive survival”. Significant differences were analyzed using one-way ANOVA with Sidak’s multiple comparison tests in **e** and two-way ANOVA with Tukey’s multiple comparison tests in **g**, **h**; **p < 0.01, ***p < 0.005, ****p < 0.001. Data are shown as mean ± SD of experimental replicates; *n* = 6 in **e** and *n* = 4–6 in **g**, **h**, representing separate wells.

We next explored the molecular mechanisms underlying STAT3-mediated adaptive survival of *ALK*-rearranged lung cancer cells by DNA microarray analysis in YHO-1701-treated cells. Focusing on anti-apoptotic genes, we identified *BCL2L1* (encoding the anti-apoptotic protein, BCL-X_L_) as one of the most downregulated genes in YHO-1701-treated cells compared to the control (Fig. 4a). We confirmed the DNA microarray results by western blotting, where BCL-X_L_ abundance was significantly reduced in H3122, H2228, and A925L cells following complete pharmacological STAT3 inhibition by combined alectinib and YHO-1701 treatment (Fig. 4b) and *STAT3* knockout (sg*STAT3*) with alectinib (Fig. 4c). *BCL-X*_*L*_ knockdown using siRNA (si*BCL-X*_*L*_) significantly increased apoptosis induction (Fig. 4d) and decreased viability of *ALK*-rearranged lung cancer cells, compared to those treated with alectinib alone (Fig. 4e). Hence, BCL-X_L_ is necessary for the survival of *ALK*-rearranged lung cancer cells upon ALK inhibition.

Alternatively, *BCL-X*_*L*_ overexpression using a lentiviral expression vector significantly decreased apoptosis induction in *ALK*-rearranged lung cancer cells treated with alectinib and YHO-1701 (Fig. 4f). The combinational efficacy of YHO-1701 was abolished by *BCL-X*_*L*_ overexpression (Fig. 4g, h), demonstrating that BCL-X_L_ influences STAT3-mediated adaptive survival and that its downregulation is necessary for inducing apoptosis in adaptive *ALK*-rearranged lung cancer cells and eradicating residual tumors. Even after long-term incubation (10 days) with ALK-TKIs (alectinib, ceritinib, lorlatinib, and brigatinib) and YHO-1701, *BCL-X*_*L*_-overexpressing cells could not be eliminated (Fig. 4i and Supplementary Fig. 6). Taken together, these results indicate that STAT3 helps cells evade apoptosis upon ALK inhibition by promoting *BCL-X*_*L*_ expression (Fig. 4j).

### STAT3 activity is maintained by multiple upstream factors upon ALK inhibition

STAT3 activity was restored and stably maintained upon initial ALK-TKIs (alectinib, ceritinib, lorlatinib, and brigatinib) treatment in the absence of ALK signaling (Fig. 1g and Supplementary Fig. 3a), characterizing the treatment-induced adaptive survival of *ALK*-rearranged lung cancer cells. STAT3 is typically activated in cancer cells via various upstream factors such as cytokines and growth factors. For instance, increased interleukin-6 (IL-6) expression commonly activates the JAK/STAT3 pathway, while autocrine stimulation of growth factor receptors, such as EGFR, also activates STAT3^33^. Furthermore, fibroblast growth factor receptor (FGFR) and IL-6 signaling work in a concerted manner to activate STAT3 following erlotinib treatment in *EGFR*-mutant lung cancer cells, contributing to the resistance of drug-treated “oncogene-addicted cancer cells”^35^. We focused on changes in cytokine levels and physical interactions of STAT3 with growth factor or cytokine receptors. Only a slight (1.3–2.0 fold) increase was observed in IL-6 levels after the treatment with alectinib, which may have been sufficient to maintain the baseline activity of STAT3 (Supplementary Fig. 7a). Increased physical interactions were detected between STAT3 and several growth factor receptors (FGFR1/2/3/4 or MET), or cytokine receptors (glycoprotein 130 and its binding protein JAK1/2, tyrosine kinase 2; Supplementary Fig. 7b). To determine the effects of the increased interactions on STAT3 activation, knockdown of *FGFRs* (si*FGFR1/2/3/4*), *MET* (si*MET*), and *JAKs* (si*JAK1/2*) was performed. *FGFR4, MET, JAK1*, and *JAK2* knockdown in H3122 cells, *FGFR1, MET, JAK1*, and *JAK2* knockdown in H2228 cells, and *FGFR3* and *JAK1* knockdown in A925L cells suppressed the phosphorylation of STAT3 recovered under the alectinib treatment (Supplementary Fig. 7c). These findings suggest that STAT3 activity is maintained via multiple upstream factors upon ALK inhibition. Moreover, the combined effects exerted by the inhibitors, including BGJ-398 (pan FGFR inhibitor), JNJ38877605 (MET specific inhibitor), and ruxolitinib (pan JAK inhibitor), were limited compared to those induced by YHO-1701 (Supplementary Fig. 7d, e). “Direct STAT3 inhibition” may be more effective for suppressing treatment-induced STAT3 activation and the subsequent adaptive survival of *ALK*-rearranged lung cancer cells (Supplementary Fig. 7f).

### Acquired resistant *ALK*-rearranged lung cancer cells are less dependent on STAT3 activity

To examine the roles of STAT3 in acquired resistance of *ALK*-rearranged lung cancer, we evaluated STAT3 activity and effects of the combined treatment on acquired resistant *ALK*-rearranged cells. We have established alectinib-acquired resistant A925L AR cells from leptomeningeal carcinomatosis model in vivo as previously described^16^. The A925L AR cells were moderately resistant to alectinib compared with A925L parental cells (Supplementary Fig. 8a; IC_50_ A925L AR, 1003.7 nmol/L; IC_50_ A925L, 167.0 nmol/L). No known *ALK* resistance mutations at I1171, F1174, R1192, L1196, L1198, G1202, G1206, G1269, or R1275 were detected, and the expression of epithelial-to-mesenchymal transition markers, such as E-cadherin and N-cadherin, remained similar between A925L AR and A925L parental cells^16^. Kinetic analysis revealed that while the phosphorylation of STAT3 in A925L parental cells was restored within 24 h, it was suppressed by alectinib without reactivation in A925L AR cells (Supplementary Fig. 8b). Meanwhile, combined alectinib and YHO-1701 treatment could not induce apoptosis in A925L AR cells (Supplementary Fig. 8c).

These results suggest that the survival of acquired resistant *ALK*-rearranged cells is less dependent on STAT3 activity, and that STAT3-targeted combined treatment is more effective for adaptive resistance to initial ALK-TKI treatment.

### Combined YHO-1701 and alectinib treatment suppresses tumor regrowth in the *ALK*-rearranged lung cancer xenograft model

**Fig. 5:**
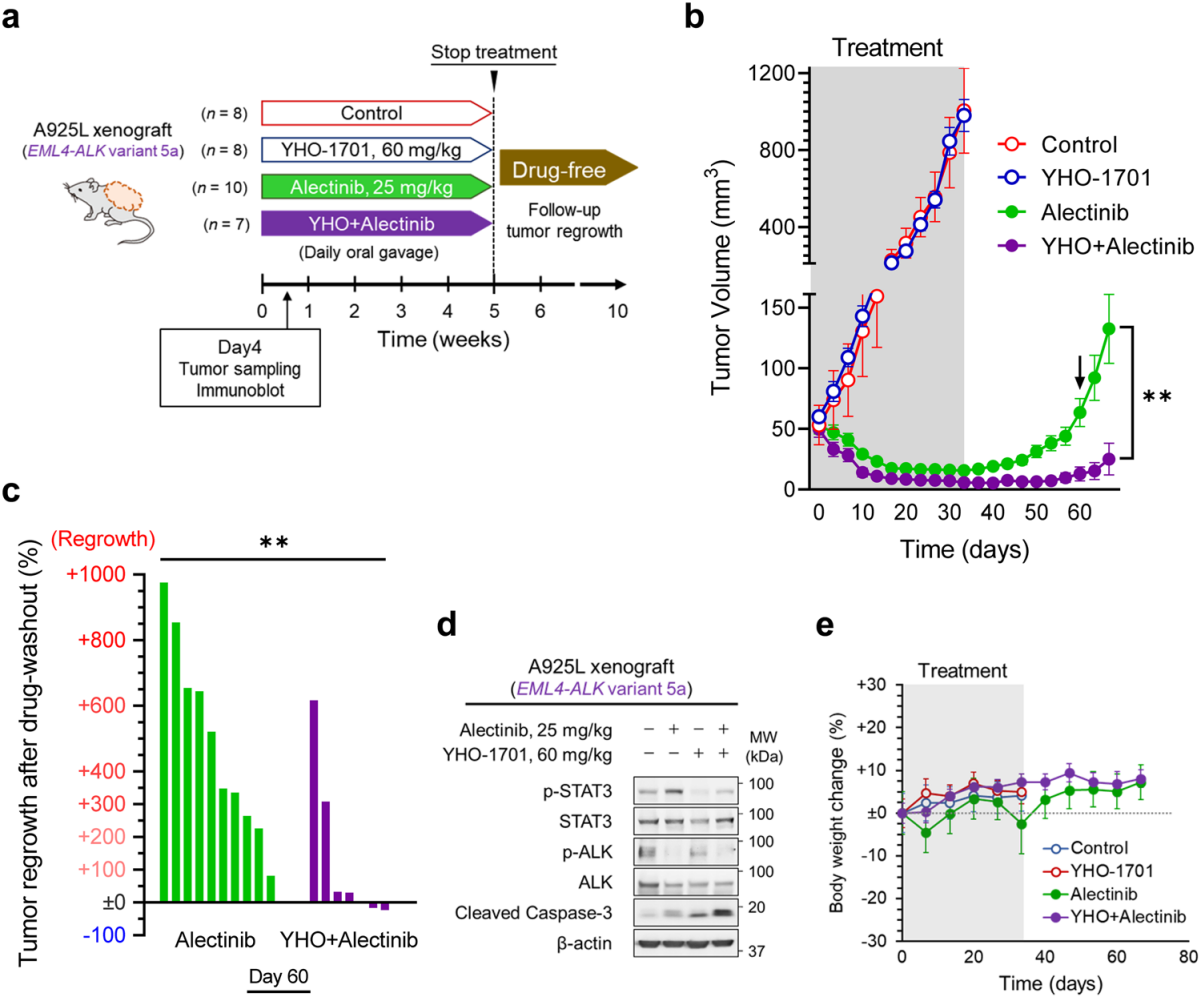
Combined YHO-1701 and alectinib treatment suppresses tumor regrowth in the *ALK*-rearranged lung cancer xenograft model. **a** Schematic representation of the in vivo experimental protocol. **b** Tumor volumes in mice bearing A925L xenografts treated with vehicle (control: *n* = 8), YHO-1701 (60 mg/kg: *n* = 8), alectinib (25 mg/kg: *n* = 10), or the combination of YHO-1701 (60 mg/kg) and alectinib (25 mg/kg) (*n* = 7). **c** Percentage changes in tumor regrowth after treatment cessation on day 60 (indicated by an arrow in **b**) in the individual A925L xenografts treated with alectinib or the combination for 5 weeks. **d** Protein expression in lysates extracted from A925L xenografts treated as indicated for 3 days. **e** Percentage body weight changes in mice. Significant differences were analyzed using two-tailed Student’s *t-*tests; **p < 0.01. Data are shown as mean ± SEM of experimental replicates; *n* = 7–10 representing separate tumors in **b** and *n* = 4–5 representing the number of mice in **e**.

We next validated the in vitro findings in vivo using cell line-derived xenograft model of *ALK*-rearranged lung cancer (Fig. 5a). After initiating treatment in mice bearing A925L xenografts, rapid tumor regression was observed in both the alectinib and alectinib/YHO-1701 combination groups. The residual tumors did not regrow during the 5 week treatment period, indicating stable residual disease maintained by adaptive survival mechanisms without acquisition of fully resistant phenotypes. However, after ceasing treatment, the xenografts in the alectinib-treated group started to regrow within 2 weeks, resulting in rapid tumor regrowth, whereas xenografts in the alectinib/YHO-1701 combination group maintained regression with near eradication (Fig. 5b, c).

Western blots revealed that YHO-1701 suppressed alectinib-induced STAT3 activation and markedly induced apoptosis in A925L xenografts (Fig. 5d). Importantly, alectinib/YHO-1701 combination treatment was well tolerated without any associated toxicity for the entire 10 weeks experimental period (Fig. 5e). These results indicate that the combined treatment of a new STAT3 inhibitor YHO-1701 with an ALK-TKI may have promising antitumor activities and potential for tumor eradication of *ALK*-rearranged lung cancer.

## Discussion

This study illustrated that STAT3 mediates treatment-induced adaptive survival of *ALK*-rearranged lung cancer cells through transcriptional regulation of apoptosis, identifying STAT3 as a promising therapeutic target of the combination therapy aimed at tumor eradication.

Aberrant STAT3 activation has been observed in various cancer types, including lung, breast, colon, liver, pancreas, head and neck, kidney, and prostate cancers, as well as multiple myeloma and plays crucial roles in tumorigenesis by promoting cell proliferation, survival, invasion, metastasis, angiogenesis, and suppression of antitumor immunity^33–37^. In the present study, we found that STAT3 activity was restored and maintained upon the initial treatment with ALK-TKIs (alectinib, ceritinib, lorlatinib, and brigatinib) in the absence of ALK and its downstream signaling. STAT3 inhibition (both genetic and pharmacological) significantly reduced cell survival by enhancing ALK inhibition-induced apoptosis, indicating that treatment-induced survival of adaptive cells upon ALK inhibition is predominantly dependent on STAT3 activity. Main limitation of the present study is that it’s unclear if STAT3 and ALK co-inhibition will have clinical relevance. Further examinations are required to obtain some corroborating evidence from clinical samples or patient-derived xenograft in vivo experiments. We elucidated molecular mechanisms underlying STAT3-mediated adaptive survival of *ALK*-rearranged lung cancer cells. Regarding apoptosis evasion upon ALK inhibition, we identified anti-apoptotic factor BCL-X_L_ as an effector of STAT3, and the combined effects of STAT3 inhibition were characterized by treatment-induced STAT3 dependence followed by transcriptional downregulation of *BCL-X*_*L*_. However, considering that other anti-apoptotic genes (e.g., *BIRC5* [survivin], *BCL2*, and *MCL1*), factors associated with cell proliferation/cycle (e.g., *MYC* and *CCND1* [cyclin D1]), and cancer stemness are coordinately involved in STAT3-mediated adaptive survival of *ALK*-rearranged lung cancer cells, further investigations are required to elucidate the detailed mechanism. Additionally, the molecular mechanisms by which STAT3 activity is maintained upon ALK inhibition require further elucidation. In addition to the upstream factors, including cytokine (IL-6) and growth factor receptors (FGFR1/2/3/4 and MET), loss-of-function mutation or treatment-induced loss of “negative regulators”, such as SOCS, SHP-1, PIAS, and PTPRT, which have been reported in various cancer types^38–40^, may also contribute to aberrant STAT3 activation. Hence, “direct STAT3 inhibitors” could effectively suppress treatment-induced and multiple factors-mediated STAT3 activation as well as subsequent adaptive cancer cell survival.

Considering our findings, a clinical trial is warranted to assess the efficacy of the new combination therapy co-targeting ALK and STAT3 in *ALK*-rearranged lung cancer. To date, several orally bioavailable STAT3 inhibitors, such as OPB31121, OPB-51602, and C188-9, have been developed and evaluated in phase I clinical trials (NCT00955812, NCT01184807, and NCT01344876), with various intolerable adverse events revealed^41,42^. Thus, STAT3 has been often considered an “undruggable” target. Moreover, the STAT family has highly homogenous structures, and therefore developing highly selective STAT3 inhibitors without unwanted side effects is a challenge. Indeed, OPB-31121 and C188-9 have been reported to inhibit STAT1 and STAT5 in addition to STAT3^43,44^. YHO-1701 is a STAT3-SH2 domain inhibitor developed through structural optimization of its lead compound, STX-0119^34^. We ensured promising target-selectivity of YHO-1701 for STAT3 and less non-specific toxicity in vitro assay. Previous studies employing several human cancer cell line-derived xenograft models, including the head and neck cancer cell line SAS-xenograft, melanoma cell line SEKI-xenograft, and cutaneous T-cell lymphoma cell line HH-xenograft, have reported that orally administered YHO-1701 induced significant antitumor effects without increasing systemic toxicity^34^. While YHO-1701 has demonstrated low non-specific toxicity, long-term inhibition of STAT3 may cause systemic toxicity given the crucial role of STAT3 as an intracellular transcription factor. Thus, optimizing the treatment schedules for reducing toxicity requires further investigations, including transient combined treatment of YHO-1701 during the early phase of therapy to induce initial apoptosis. Given the toxicity observed with other STAT3 inhibitors, further assessment regarding the off-target toxicity is warranted prior to clinical trials. Moreover, work by Bivona and colleagues has shown that genetic activation of STAT3 partially rescued *ALK*-rearranged lung cancer cells from the effects of ALK inhibitor treatment, with greater dependence on the RAS-MAPK pathway^45^. Interestingly, in our examination, different sensitivities to MEK or STAT3 inhibitor were observed between the types of *EML4-ALK* variants (variant 1: H3122, variant 3a/b: H2228, and variant 5a: A925L; Supplementary Fig. 9a–c). Therefore, further considerations are required on case selection for the STAT3-targeted treatment considering not only the toxicities but also the types of *EML4-ALK* fusion variants.

In summary, the mechanisms underlying treatment-induced adaptive cancer cell survival in residual tumors of *ALK*-rearranged lung cancer are predominantly dependent on STAT3 activity and subsequent transcriptional regulation of apoptosis. This was evidenced by the application of combined treatment co-targeting ALK and STAT3 with a new and highly selective STAT3 inhibitor, YHO-1701, that effectively suppressed the adaptive survival of *ALK*-rearranged lung cancer cells by enhancing apoptosis induction via transcriptional downregulation of the anti-apoptotic factor *BCL-X*_*L*_. This treatment regimen also significantly suppressed tumor regrowth with near tumor remission in xenograft study. Our results indicate that inhibition of the adaptive survival via STAT3 combined with an ALK-TKI may improve the outcomes of *ALK*-rearranged lung cancer. Furthermore, the safety and efficacy of this combination therapy must be evaluated and validated in the clinical trials.

## Methods

### Cell culture and reagents

H3122 (*EML4-ALK* variant 1 E13; A20) cells were provided by Dr. Jeffrey A. Engelman (Novartis Institutes for BioMedical Research, Cambridge, MA, USA). H2228 (*EML4-ALK* variant 3a/b E6; A20) cells were purchased from American Type Culture Collection (ATCC, Manassas, VA, USA). The A925L (*EML4-ALK* variant 5a E2; A20) cell line was established from a surgical specimen obtained from a male Japanese patient (T2N2M0, stage IIIA) and provided by Drs. Fumihiro Tanaka and Hidetaka Uramoto (University of Occupational and Environmental Health, Fukuoka, Japan). Human lung embryonic fibroblast cell lines, MRC-5 and IMR-90 were obtained from RIKEN Cell Bank (Ibaraki, Japan). All cell lines were cultured in RPMI 1640 medium (Gibco, Waltham, MA, USA) supplemented with 10% FBS (Gibco) and 1% penicillin–streptomycin (FUJIFILM Wako, Osaka, Japan) in a humidified CO_2_ incubator at 37 °C. All cells were passaged for less than 3 months from frozen early passage stocks and regularly screened for *Mycoplasma* infection using MycoAlert^TM^ Mycoplasma Detection Kit (Lonza, Basel, Switzerland). Cell lines were authenticated by short tandem repeat analysis at the National Institute of Biomedical Innovation (Osaka, Japan). Alectinib was provided by Chugai Pharmaceutical Co., Ltd. (Tokyo, Japan) and YHO-1701 by Yakult Pharmaceutical Industry Co., Ltd. (Tokyo, Japan). Ceritinib, lorlatinib, brigatinib, infigratinib (BGJ-398), JNJ-38877605, ruxolitinib and trametinib were purchased from Selleck Chemicals (Houston, TX, USA). Each compound was separately dissolved in dimethyl sulfoxide (DMSO; FUJIFILM Wako) at a concentration of 10 mM for the cell culture experiments.

### Receptor binding study of YHO-1701

To clarify the affinities of YHO-1701 for various receptors, including ion channels and transporters, the inhibition ratios of the binding between each receptor and its specific ligand were evaluated. YHO-1701 (1 and 10 µmol/L), replacement substance, positive substance, and tracer and receptor solutions were mixed and incubated under the conditions listed in Supplementary Table 3. Liquid scintillator (PICO-FLUORTM PLUS) was then added, and the radioactivity was determined using a liquid scintillation counter (measured time: 2 min). Inhibition ratios were calculated from “100 − binding ratio”; Binding ratio: [(*B* − *N*)/(*B*0 − *N*)] × 100 (%); B: Bound radioactivity in the presence of the test substance (individual value); B0: Total bound radioactivity in the absence of the test substance (mean value); N: Non-specific bound radioactivity (mean value).

### Docking study of YHO-1701

YHO-1701 docking studies were carried out with Molecular Operating Environment (MOE) 20190102 software (Chemical Computing Group, Montreal, Canada). Three-dimensional STAT3 and STAT1 homodimer structures were obtained from protein data bank (PDB): STAT3 (PDB Id: 1BG1) and STAT1 (PDB Id: 1BF5). The structures of phospho-Tyr linkers for STAT1 (aa700–710: GpY^701^IKTELISVS) and STAT3 (aa702–716: APpY^705^LKTKFICVTPF) were extracted from the crystal structure of each homodimer. During docking analysis, the structure was hydrogenated using the Protonate 3D module. After assigning partial charges using an all-atom force field combining Amber10 (ref. ^46^) and extended Hückel theory (EHT)^47^, hydrogen atoms were minimized, and DNA strands were subsequently removed. The Alpha Site Finder module^48^ was used to define a ligand-binding site targeting the Src homology 2 (SH2) domains. YHO-1701 generated by the stochastic search method was docked on the binding site. Docked poses were optimized by Amber 10: EHT force field and then ranked according to the generalized-born volume integral/weighted surface area scoring function^49^, which estimates the free energy of ligand binding from a particular angle.

### STAT alpha screen of YHO-1701

Alpha-based binding assays were performed as described previously^34^. Biotinylated recombinant proteins (STAT3, STAT1, and Grb2) were incubated for 90 min with YHO-1701 and fluorescein isothiocyanate **(**FITC)-labeled phospho-Tyr peptides and mixed with streptavidin-coated donor and anti-FITC acceptor beads simultaneously, before detection at 570 nm using an EnVision® Xcite Multilabel Reader (PerkinElmer, Waltham, MA). IC_50_ values for STAT3 were calculated using a dose-response curve (GraphPad Prism 8).

### Western blotting and antibodies

Cells were washed with PBS (Gibco) and lysed on ice using cell lysis buffer (Cell Signaling Technology, Danvers, MA, USA) supplemented with a protease and phosphatase inhibitor cocktail (P8340 and P0044; Sigma-Aldrich Corporation, St. Louis, MO, USA), and the cell extracts were collected. Cytoplasmic and nuclear fractions of the lysates were separately collected using NE-PER™ Nuclear and Cytoplasmic Extraction Reagents (Thermo Fisher Scientific, Waltham, MA, USA) according to the manufacturer’s instructions. Equal amounts of proteins (20 µg) were electrophoresed on polyacrylamide gels (Mini-PROTEAN® TGX™ Precast Gels: Bio-Rad, Hercules, CA, USA) and transferred to polyvinylidene difluoride membranes (Immun-Blot® PVDF Membrane; Bio-Rad). The membranes were then incubated with StartingBlock™ T20 (TBS) Blocking Buffer (Thermo Fisher Scientific) for 1 h at room temperature, primary antibodies for phospho-STAT3 (Tyr705, #9131), STAT3 (#4904), phospho-ALK (Tyr1604, #3341), ALK (#3633), phospho-ERK1/2 (Thr202/Tyr204, #4370), ERK1/2 (#4695), phospho-AKT (Ser473, #4060), AKT (#9272), BCL-X_L_ (#2764), cleaved caspase-3 (#9664), cleaved PARP (#5625), FGFR1 (#9740), FGFR2 (#11835), FGFR3 (#4574), FGFR4 (#8562), MET (#8198), JAK1 (#3344), JAK2 (#3230), Tyk2 (#9312), gp130 (#3732), EGFR (#4267), IGF-1R (#9750), and β-actin (#4970) (1:1,000 dilution; Cell Signaling Technology) overnight at 4 °C, and horseradish peroxidase-conjugated secondary antibody (#7074) (1:2000 dilution; Cell Signaling Technology) for 1 h at room temperature. All antibodies were diluted with 5% (w/v) BSA (Sigma-Aldrich)/TBS with 0.1% (v/v) TWEEN® 20 (TBS-T; Sigma-Aldrich), and the membranes were washed with TBS-T between each step (three times, 10 min each). Immunoreactive bands were visualized using SuperSignal™ West Dura Extended Duration Substrate (Thermo Fisher Scientific). Chemiluminescence signals were measured using a FUSION-SOLO Chemiluminescence Imaging System (Vilber Lourmat, Marne-la-Vallée, France). All blots were derived from the same experiment and were processed in parallel.

### Cell viability assay

Cells (5000 cells/well) were cultured in 96-well plates overnight and incubated with the indicated compounds for 72 h. Cell viability was quantified using MTT assay (Sigma-Aldrich). Absorbance was measured using an iMark™ Microplate Absorbance Reader (Bio-Rad). The percentage of cell viability was determined relative to untreated controls or baseline cells. IC_50_ values were calculated using a non-linear regression model with a sigmoidal dose-response curve using GraphPad Prism 8 (GraphPad Software, La Jolla, CA, USA).

### Apoptosis assay

Cells (5000 cells/well) were cultured in 96-well plates overnight and incubated with the indicated compounds for 72 h. Apoptosis was quantified using Caspase-Glo® 3/7 Assay (Promega, Madison, WI, USA) according to the manufacturer’s instructions. Luminescence was measured using a Fluoroskan Ascent™ FL Microplate Fluorometer and Luminometer (Thermo Fisher Scientific). Cell viability was also quantified simultaneously using the CellTiter-Glo® 2.0 Cell Viability Assay (Promega). Caspase 3/7 levels were normalized against cell viability.

### Colony formation assay

Cells (2.0 × 10^5^ cells/well in 6-well plates) were cultured overnight and incubated with compounds (alectinib, ceritinib, lorlatinib, brigatinib, YHO-1701, infigratinib [BGJ-398], JNJ-38877605, and ruxolitinib) for 10 days. Viable cells were fixed with 100% methanol (FUJIFILM Wako) for 5 min at room temperature, stained with crystal violet solution (Sigma-Aldrich), and washed in water three times.

### Immunofluorescence staining

Cells cultured in chamber slides were fixed with ice-cold 100% methanol (FUJIFILM Wako) for 10 min at −20 °C. The cells were then permeabilized with 0.25% (v/v) Triton™ X-100 (Sigma-Aldrich) diluted in PBS, for 10 min, followed by blocking with 5% (w/v) BSA/PBS for 30 min at room temperature. Cells were incubated with anti-STAT3 antibody (#4904, 1:1000; Cell Signaling Technology) overnight at 4 °C and Alexa Fluor 488-conjugated secondary antibody (#4412, 1:1000; Cell Signaling Technology) for 1 h at room temperature. All antibodies were diluted with 5% (w/v) BSA/PBS. The chamber slides were washed with PBS between each step (three times, 5 min each). Nuclei were counterstained with DAPI (4′,6-diamidino-2-phenylindole) (VECTASHIELD® Antifade Mounting Medium with DAPI, Vector Laboratories, Burlingame, CA, USA). For quantification of STAT3 nuclear translocation, we counted the STAT3 nuclear localized cells, which are cells with completely positively stained for the nucleus. The percentage of STAT3 nuclear localized cells was calculated among total cells in 4 randomly selected fields.

### Transfection with siRNAs

*Silencer*® Select siRNAs for *STAT3* (s744, s745), *BCL2L1* (encoding BCL-X_L_; s1920, s1922), *FGFR1* (s5164), *FGFR2* (s5175), *FGFR3* (s534558), *FGFR4* (s5178), *MET* (s8700), *JAK1* (s7646), and *JAK2* (s7649), and *Silencer*® Select Negative Control siRNA #1 (#4390843) were purchased from Thermo Fisher Scientific. Cells were transfected with siRNAs by reverse transfection using Lipofectamine™ RNAiMAX Transfection Reagent (Invitrogen, Waltham, MA, USA) according to the manufacturer’s instructions. Gene knockdown was confirmed by western blotting.

### Generation of shRNA- and cDNA-expressing stable cell lines

The short hairpin RNA (shRNA)-expressing lentiviral vectors (pSIH1-puro-STAT3 shRNA: #26596, pSIH1-puro-control shRNA: #26597) (Addgene, Watertown, MA, USA) and cDNA-expressing lentiviral vectors (pCDH-puro-BCL-X_L_: #46972 [Addgene], pCDH-CMV-MCS-EF1-Puro: #CD510B-1 [System Biosciences, Palo Alto, CA, USA]) were used to stably express shRNA or cDNA, respectively. To generate lentiviruses, HEK 293T/17 cells were transfected with lentiviral vectors and packaging plasmids (Lentiviral High Titer Packaging Mix: Takara Bio, Shiga, Japan) using *Trans*IT®-293 transfection reagent (Mirus Bio, Madison, WI, USA). On the following day, the medium was replaced with fresh growth medium, and lentivirus-containing supernatants were harvested and concentrated by centrifugation using a Lenti-X™ Concentrator (Takara Bio). To establish shRNA- and cDNA-expressing stable cell lines, the cells were transduced with lentiviral particles using 1 μg/mL Polybrene® (Santa Cruz Biotechnology, Dallas, TX, USA) and then selected with 2 μg/mL puromycin (Sigma-Aldrich) for 10–14 days. Stable gene knockdown was confirmed by western blotting.

### ELISA

Human IL-6 ELISAs (R&D systems) were performed according to manufacturer’s instructions. Conditioned media from each cell lines was collected after 48 h culture in the presence of the indicated drug concentration. Absorbance was measured using an iMark™ Microplate Absorbance Reader (Bio-Rad).

### CRISPR-Cas9 gene editing

To generate cell lines stably expressing Cas9 nuclease and small guide RNAs (sgRNAs), the Cas9 and sgRNA vectors (LentiV_Cas9_puro: #108100, pSLQ1371-sgSTAT3-1: #121425, non-targeting control gRNA: #80236) (Addgene) were packaged into lentiviral particles and concentrated as described in “shRNA- and cDNA-expressing stable cell lines”. Cas9-expressing cell lines were generated with the Cas9 nuclease-expressing lentiviral particles, and subsequent gene knockouts were obtained by additional transduction with the sgRNA lentiviral particles. CRISPR-KO screening was performed using Dharmacon Edit-R^TM^ synthetic sgRNA libraries (Horizon Discovery, Waterbeach, UK), according to the manufacturer’s instructions.

### Microarray analysis

Microarray gene expression profiles were generated using GeneChip^TM^ WT PLUS Reagent Kit (Thermo Fisher Scientific, Waltham, MA, USA) according to the manufacturer’s recommendations by GeneticLab Co., Ltd. (Sapporo, Japan). Gene expression signal values were extracted from the CEL files and normalized according to the guanine cytosine count normalization-signal space transformation-robust multichip analysis (GCCN-SST-RMA) algorithms. The signal values were log_2_ transformed.

### Xenograft study

Specific pathogen-free male SHO mice (Crlj:SHO-*Prkdc* ^*scid*^*Hr* ^*hr*^) were purchased from Charles River Laboratories Japan, Inc. (Kanagawa, Japan). A925L cells (5.0 × 10^6^ cells) suspended in 50% (v/v) Matrigel® (Corning, New York, NY, USA)/Hanks’ balanced salt solution (Gibco) were subcutaneously injected into both flanks of 6-week-old male mice. When the average tumor volume reached approximately 100 mm^3^, the mice were randomized into four groups and treated with alectinib (25 mg/kg/day), YHO-1701 (60 mg/kg/day), alectinib and YHO-1701, or control, by oral gavage for 5 days/week for 5 weeks. Alectinib was resolved in 15% (v/v) polyethylene glycol (PEG) 400 (Wako), 15% (v/v) (2-Hydroxypropyl)-β-cyclodextrin (Sigma-Aldrich), Cremophor® EL (Sigma-Aldrich), and 20 mmol/L HCl (Wako). YHO-1701 was resolved in 10% (w/v) polyvinylpyrrolidone K 30 (FUJIFILM Wako)/ PEG 400. Tumor size and body weight were measured twice per week, and tumor volumes (mm^3^) were calculated using the following formula: 1/2 × length (mm) × (width [mm])^2^. All animal experiments were performed in strict accordance with the Guide for the Care and Use of Laboratory Animals from the Ministry of Education, Culture, Sports, Science, and Technology, Japan. The study protocol was approved by the Ethics Committee on the Use of Laboratory Animals and the Advanced Science Research Center, Kanazawa University, Kanazawa, Japan (approval no. AP-173867).

### Statistical analysis

Continuous variables were expressed as means ± SDs or SEMs. The statistical significance of the differences was analyzed using two-tailed Student’s *t*-test and one-way or two-way analysis of variance (ANOVA) with Sidak’s or Tukey’s multiple comparison tests. *P* < 0.05 were considered statistically significant. All statistical analyses of experimental data were performed using GraphPad Prism 8 (GraphPad Software).

### Reporting summary

Further information on research design is available in the Nature Research Reporting Summary linked to this article.

## Supplementary information

Supplementary Information

REPORTING SUMMARY

## Data Availability

The microarray data used in the current study have been deposited in Gene Expression Omnibus (GEO) under the accession code GSE193858.

## Code availability

No custom code or algorithms were created.

## References

[1] Paez JG (2004). EGFR mutations in lung cancer: correlation with clinical response to gefitinib therapy. Science.

[2] Maemondo M (2010). Gefitinib or chemotherapy for non-small-cell lung cancer with mutated EGFR. N. Engl. J. Med..

[3] Soda M (2007). Identification of the transforming EML4-ALK fusion gene in non-small-cell lung cancer. Nature.

[4] Rikova K (2007). Global survey of phosphotyrosine signaling identifies oncogenic kinases in lung cancer. Cell.

[5] Lin JJ, Riely GJ, Shaw AT (2017). Targeting ALK: Precision medicine takes on drug resistance. Cancer Discov..

[6] Hida T (2017). Alectinib versus crizotinib in patients with ALK-positive non-small-cell lung cancer (J-ALEX): an open-label, randomised phase 3 trial. Lancet.

[7] Peters S (2017). Alectinib versus crizotinib in untreated ALK-positive non-small-cell lung cancer. N. Engl. J. Med..

[8] Camidge DR (2018). Brigatinib versus crizotinib in ALK-positive non-small-cell lung cancer. N. Engl. J. Med..

[9] Shaw AT (2020). First-line lorlatinib or crizotinib in advanced ALK-positive lung cancer. N. Engl. J. Med..

[10] Gainor JF (2016). Molecular mechanisms of resistance to first- and second-generation ALK inhibitors in ALK-rearranged lung cancer. Cancer Discov..

[11] Katayama R (2014). Two novel ALK mutations mediate acquired resistance to the next-generation ALK inhibitor alectinib. Clin. Cancer Res..

[12] Friboulet L (2014). The ALK inhibitor ceritinib overcomes crizotinib resistance in non-small cell lung cancer. Cancer Discov..

[13] Yoda S (2018). Sequential ALK inhibitors can select for lorlatinib-resistant compound ALK mutations in ALK-positive lung cancer. Cancer Discov..

[14] Recondo G (2020). Diverse resistance mechanisms to the third-generation ALK inhibitor lorlatinib in ALK-rearranged lung cancer. Clin. Cancer Res..

[15] Yamada T (2012). Paracrine receptor activation by microenvironment triggers bypass survival signals and ALK inhibitor resistance in EML4-ALK lung cancer cells. Clin. Cancer Res..

[16] Arai S (2020). Osimertinib overcomes alectinib resistance caused by amphiregulin in a leptomeningeal carcinomatosis model of ALK-rearranged lung cancer. J. Thorac. Oncol..

[17] Dagogo-Jack I (2020). MET alterations are a recurring and actionable resistance mechanism in ALK-positive lung cancer. Clin. Cancer Res..

[18] Isozaki H (2016). Non-small cell lung cancer cells acquire resistance to the ALK inhibitor alectinib by activating alternative receptor tyrosine kinases. Cancer Res..

[19] Crystal AS (2014). Patient-derived models of acquired resistance can identify effective drug combinations for cancer. Science.

[20] Fukuda K (2019). Epithelial-to-mesenchymal transition is a mechanism of ALK inhibitor resistance in lung cancer independent of ALK mutation status. Cancer Res..

[21] Sharma SV (2010). A chromatin-mediated reversible drug-tolerant state in cancer cell subpopulations. Cell.

[22] Bivona TG, Doebele RC (2016). A framework for understanding and targeting residual disease in oncogene-driven solid cancers. Nat. Med..

[23] Kurppa KJ (2020). Treatment-induced tumor dormancy through YAP-mediated transcriptional reprogramming of the apoptotic pathway. Cancer Cell.

[24] Arasada RR (2018). Notch3-dependent β-catenin signaling mediates EGFR TKI drug persistence in EGFR mutant NSCLC. Nat. Commun..

[25] Taniguchi H (2019). AXL confers intrinsic resistance to osimertinib and advances the emergence of tolerant cells. Nat. Commun..

[26] Wang R (2020). Transient IGF-1R inhibition combined with osimertinib eradicates AXL-low expressing EGFR mutated lung cancer. Nat. Commun..

[27] Nakagawa T (2013). EGFR-TKI resistance due to BIM polymorphism can be circumvented in combination with HDAC inhibition. Cancer Res..

[28] Tanimoto A (2017). Histone deacetylase 3 inhibition overcomes BIM deletion polymorphism-mediated osimertinib resistance in EGFR-mutant lung cancer. Clin. Cancer Res..

[29] Shaw AT (2017). Lorlatinib in non-small-cell lung cancer with ALK or ROS1 rearrangement: an international, multicenter, open-label, single-arm first-in-man phase 1 trial. Lancet Oncol..

[30] Seto T (2013). CH5424802 (RO5424802) for patients with ALK-rearranged advanced non-small-cell lung cancer (AF-001JP study): a single-arm, open-label, phase 1-2 study. Lancet Oncol..

[31] Shaw AT, Engelman JA (2014). Ceritinib in ALK-rearranged non-small-cell lung cancer. N. Engl. J. Med..

[32] Gettinger SN (2016). Activity and safety of brigatinib in ALK-rearranged non-small-cell lung cancer and other malignancies: a single-arm, open-label, phase 1/2 trial. Lancet Oncol..

[33] Johnson DE, O’Keefe RA, Grandis JR (2018). Targeting the IL-6/JAK/STAT3 signaling axis in cancer. Nat. Rev. Clin. Oncol..

[34] Nishisaka F (2020). Antitumor activity of a novel oral signal transducer and activator of transcription 3 inhibitor YHO-1701. Cancer Sci..

[35] Lee HJ (2014). Drug resistance via feedback activation of Stat3 in oncogene-addicted cancer cells. Cancer Cell.

[36] Siersbæk R (2020). IL6/STAT3 signaling hijacks estrogen receptor α enhancers to drive breast cancer metastasis. Cancer Cell.

[37] Yu H, Pardoll D, Jove R (2009). STATs in cancer inflammation and immunity: a leading role for STAT3. Nat. Rev. Cancer.

[38] Buchert M, Burns CJ, Ernst M (2016). Targeting JAK kinase in solid tumors: emerging opportunities and challenges. Oncogene.

[39] Tartaglia M (2003). Somatic mutations in PTPN11 in juvenile myelomonocytic leukemia, myelodysplastic syndromes and acute myeloid leukemia. Nat. Genet..

[40] Zhang X (2007). Identification of STAT3 as a substrate of receptor protein tyrosine phosphatase T. Proc. Natl Acad. Sci. USA.

[41] Bendell JC (2014). Phase 1, open-label, dose-escalation, and pharmacokinetic study of STAT3 inhibitor OPB-31121 in subjects with advanced solid tumors. Cancer Chemother. Pharmacol..

[42] Wong AL (2015). Phase I and biomarker study of OPB-51602, a novel signal transducer and activator of transcription (STAT) 3 inhibitor, in patients with refractory solid malignancies. Ann. Oncol..

[43] Kim MJ (2013). OPB-31121, a novel small molecular inhibitor, disrupts the JAK2/STAT3 pathway and exhibits an antitumor activity in gastric cancer cells. Cancer Lett..

[44] Bharadwaj U (2016). Small-molecule inhibition of STAT3 in radioresistant head and neck squamous cell carcinoma. Oncotarget.

[45] Hrustanovic G (2015). RAS-MAPK dependence underlies a rational polytherapy strategy in EML4-ALK-positive lung cancer. Nat. Med..

[46] Case, D. A. et al. AMBER 10 (University of California, 2008).

[47] Gerber PR, Müller K (1995). MAB, a generally applicable molecular force field for structure modelling in medicinal chemistry. J. Comput. Aided Mol. Des..

[48] Soga S, Shirai H, Kobori M, Hirayama N (2007). Use of amino acid composition to predict ligand-binding sites. J. Chem. Inf. Model..

[49] Corbeil CR, Williams CI, Labute P (2012). Variability in docking success rates due to dataset preparation. J. Comput. Aided Mol. Des..

