# Supplementary material for: STAT3 inhibition suppresses adaptive survival of ALK-rearranged lung cancer cells through transcriptional modulation of apoptosis
Source: NPJ Precis Oncol. 2022 Feb 28;6:11. doi: 10.1038/s41698-022-00254-y (PMC8885877; doi:10.1038/s41698-022-00254-y)
Supplement: Supplementary file 1 — Supplementary Information [file 41698_2022_254_MOESM1_ESM.pptx]

## Slide 1
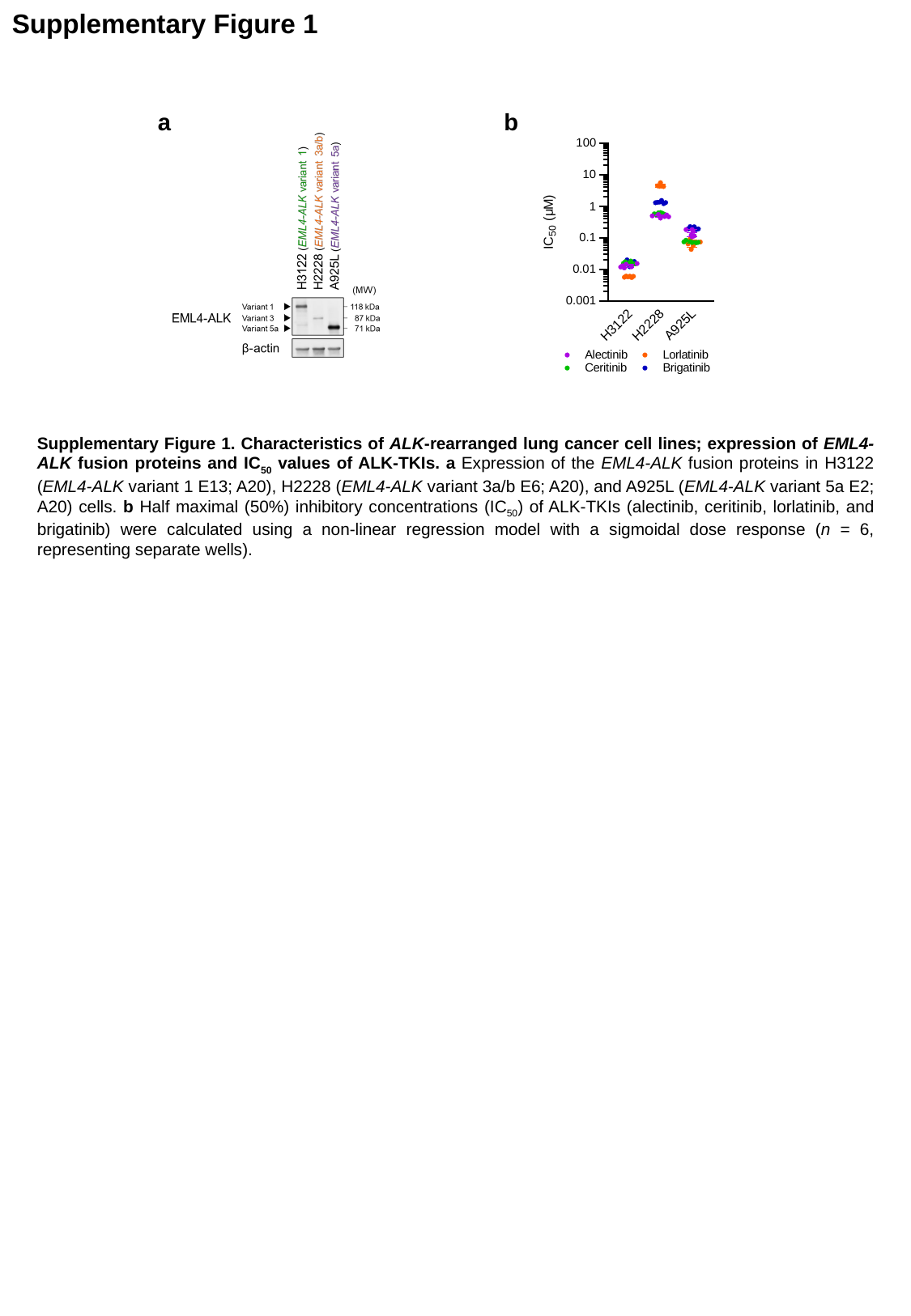

Supplementary Figure 1
a
b
Supplementary Figure 1. Characteristics of ALK-rearranged lung cancer cell lines; expression of EML4-ALK fusion proteins and IC50 values of ALK-TKIs. a Expression of the EML4-ALK fusion proteins in H3122 (EML4-ALK variant 1 E13; A20), H2228 (EML4-ALK variant 3a/b E6; A20), and A925L (EML4-ALK variant 5a E2; A20) cells. b Half maximal (50%) inhibitory concentrations (IC50) of ALK-TKIs (alectinib, ceritinib, lorlatinib, and brigatinib) were calculated using a non-linear regression model with a sigmoidal dose response (n = 6, representing separate wells).

## Slide 2
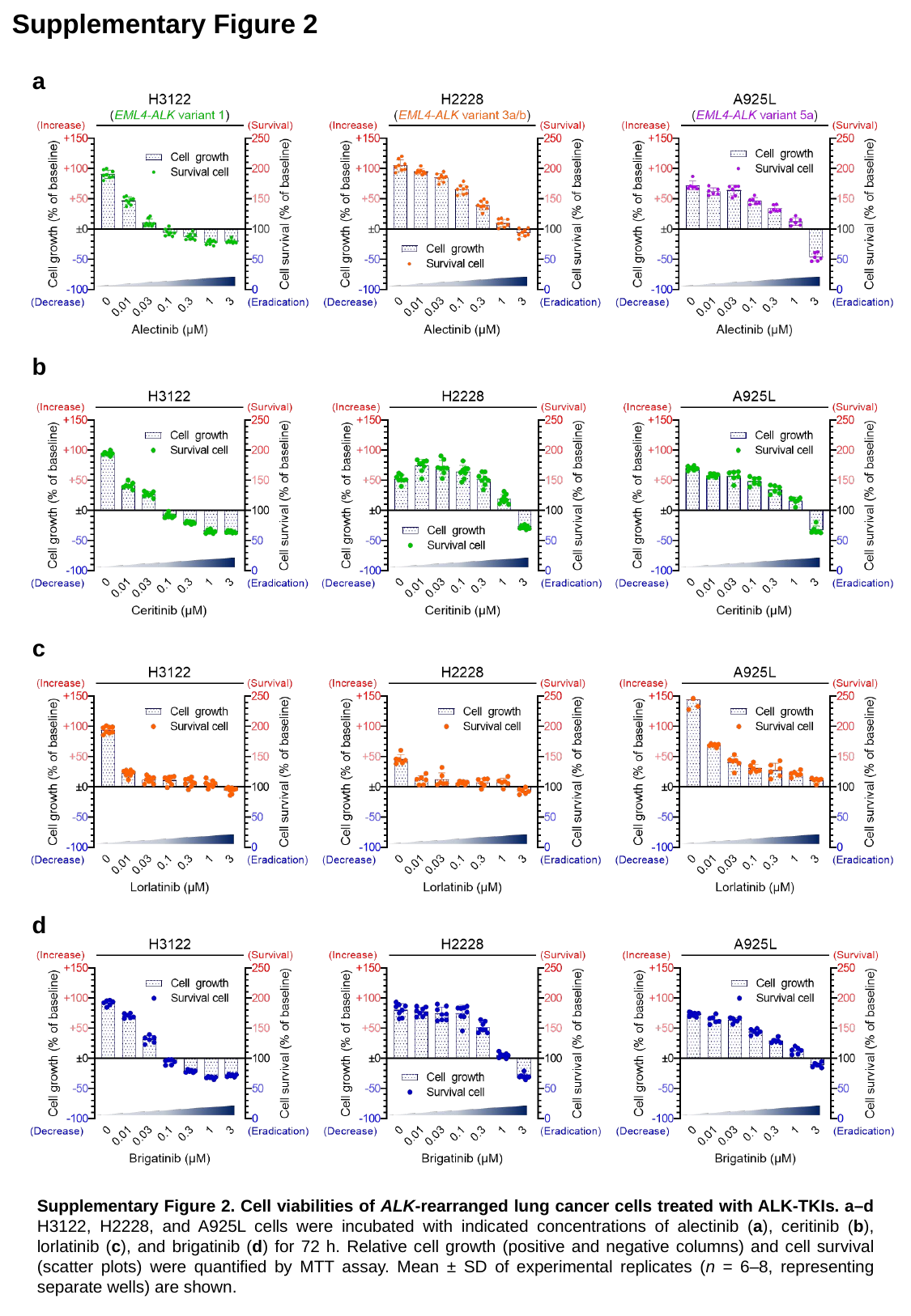

Supplementary Figure 2
a
b
c
d
Supplementary Figure 2. Cell viabilities of ALK-rearranged lung cancer cells treated with ALK-TKIs. a–d H3122, H2228, and A925L cells were incubated with indicated concentrations of alectinib (a), ceritinib (b), lorlatinib (c), and brigatinib (d) for 72 h. Relative cell growth (positive and negative columns) and cell survival (scatter plots) were quantified by MTT assay. Mean ± SD of experimental replicates (n = 6–8, representing separate wells) are shown.

## Slide 3
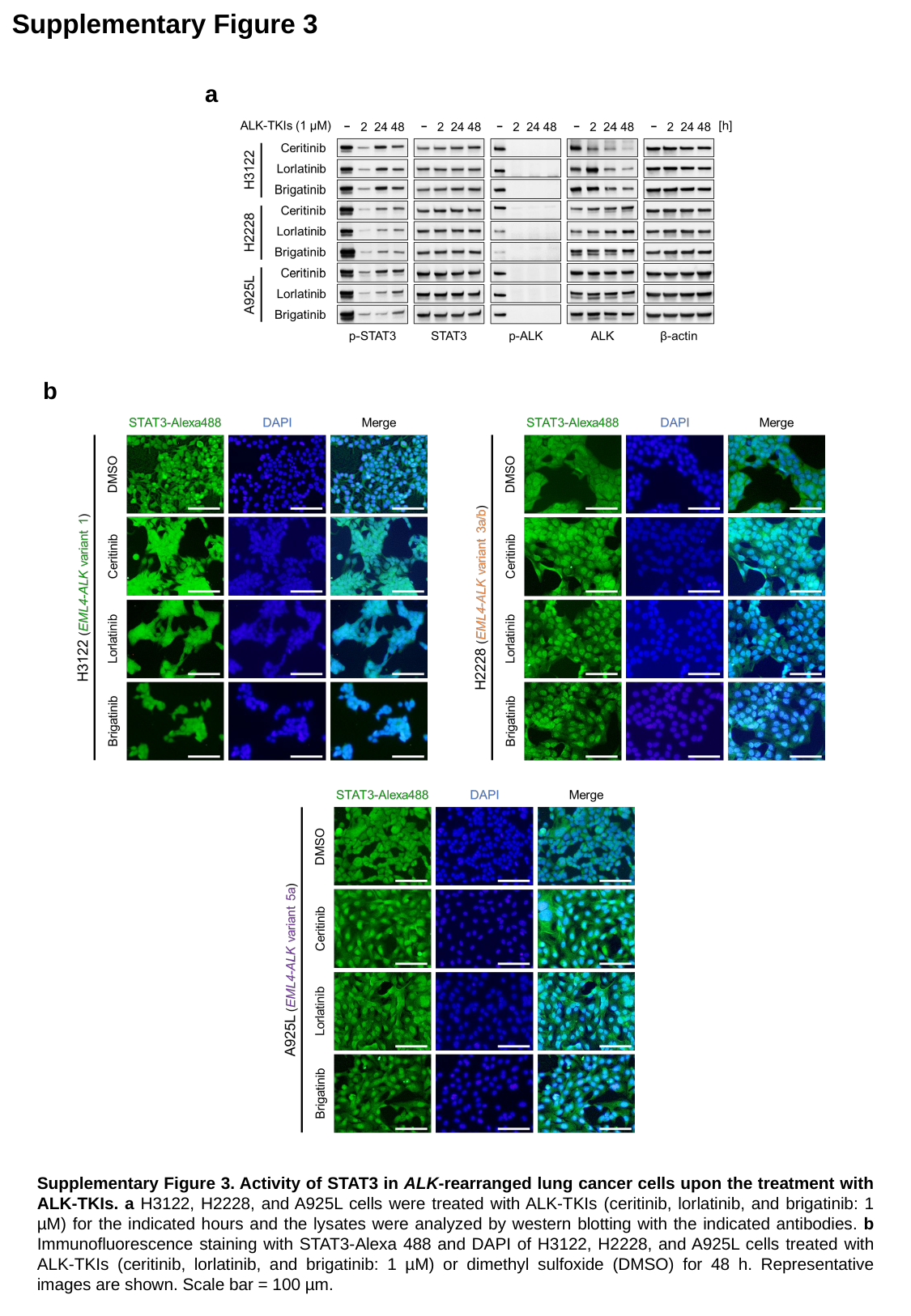

Supplementary Figure 3
a
b
Supplementary Figure 3. Activity of STAT3 in ALK-rearranged lung cancer cells upon the treatment with ALK-TKIs. a H3122, H2228, and A925L cells were treated with ALK-TKIs (ceritinib, lorlatinib, and brigatinib: 1 µM) for the indicated hours and the lysates were analyzed by western blotting with the indicated antibodies. b Immunofluorescence staining with STAT3-Alexa 488 and DAPI of H3122, H2228, and A925L cells treated with ALK-TKIs (ceritinib, lorlatinib, and brigatinib: 1 µM) or dimethyl sulfoxide (DMSO) for 48 h. Representative images are shown. Scale bar = 100 µm.

## Slide 4
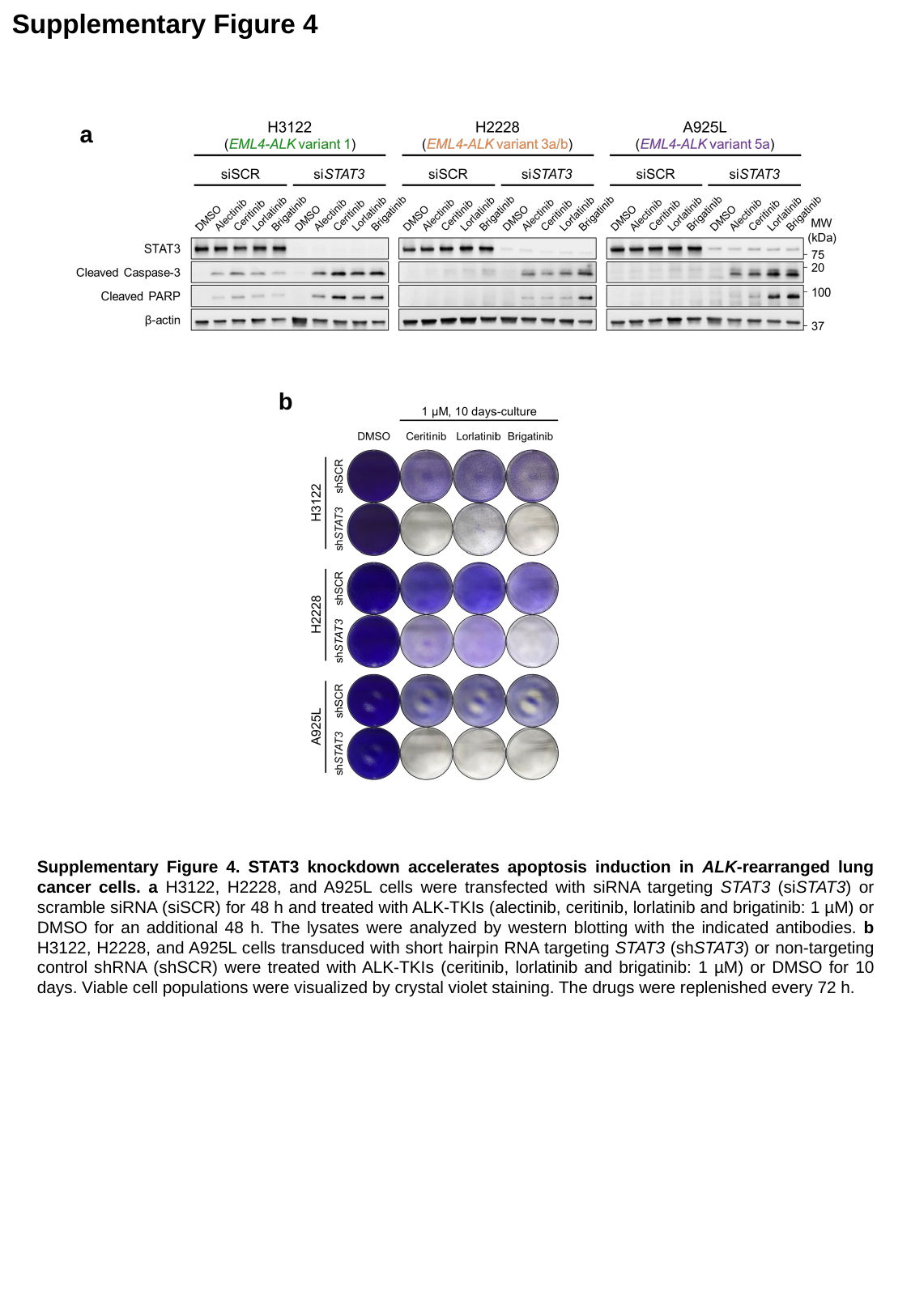

Supplementary Figure 4
a
b
Supplementary Figure 4. STAT3 knockdown accelerates apoptosis induction in ALK-rearranged lung cancer cells. a H3122, H2228, and A925L cells were transfected with siRNA targeting STAT3 (siSTAT3) or scramble siRNA (siSCR) for 48 h and treated with ALK-TKIs (alectinib, ceritinib, lorlatinib and brigatinib: 1 µM) or DMSO for an additional 48 h. The lysates were analyzed by western blotting with the indicated antibodies. b H3122, H2228, and A925L cells transduced with short hairpin RNA targeting STAT3 (shSTAT3) or non-targeting control shRNA (shSCR) were treated with ALK-TKIs (ceritinib, lorlatinib and brigatinib: 1 µM) or DMSO for 10 days. Viable cell populations were visualized by crystal violet staining. The drugs were replenished every 72 h.

## Slide 5
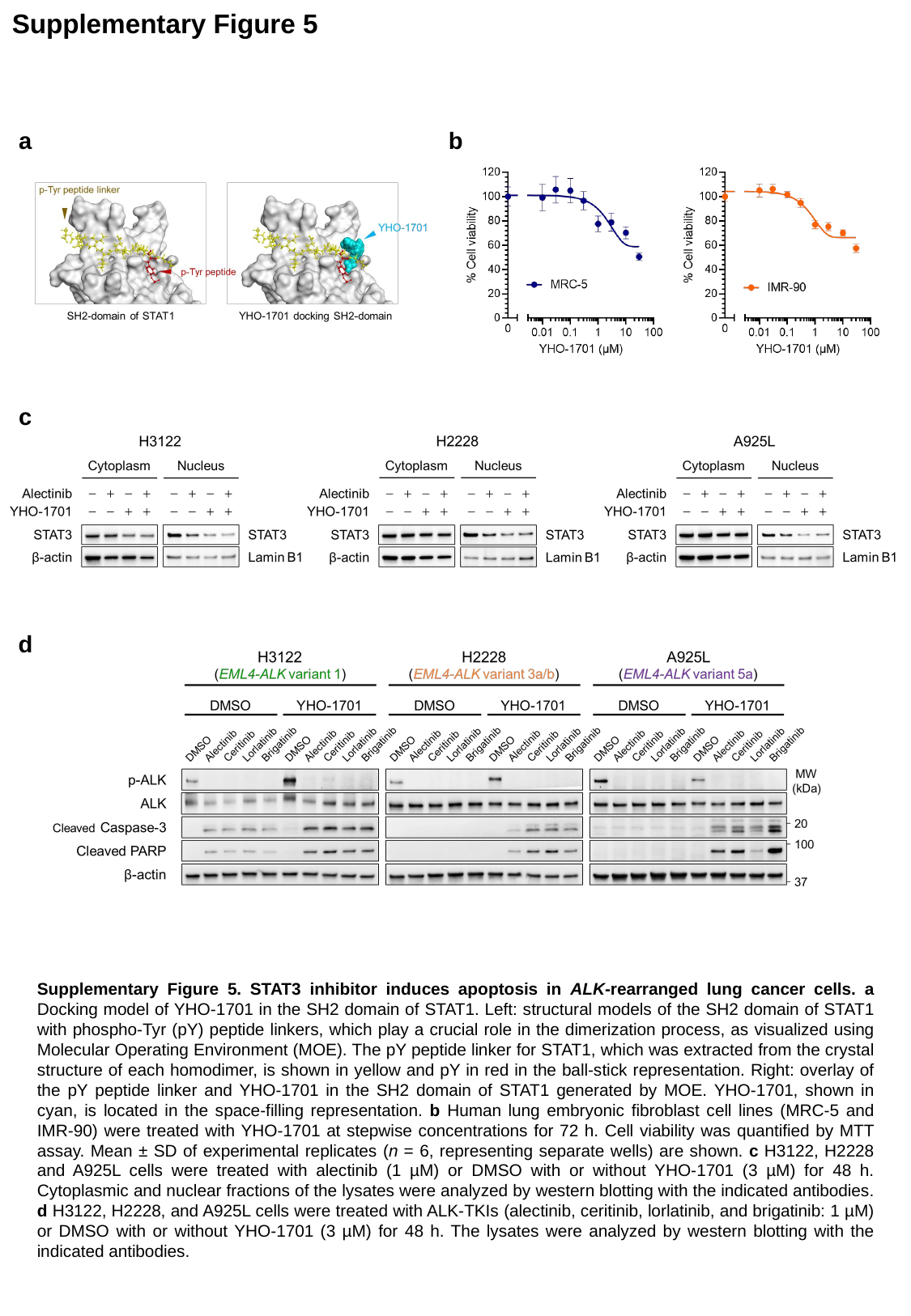

Supplementary Figure 5
a
b
c
d
Supplementary Figure 5. STAT3 inhibitor induces apoptosis in ALK-rearranged lung cancer cells. a Docking model of YHO-1701 in the SH2 domain of STAT1. Left: structural models of the SH2 domain of STAT1 with phospho-Tyr (pY) peptide linkers, which play a crucial role in the dimerization process, as visualized using Molecular Operating Environment (MOE). The pY peptide linker for STAT1, which was extracted from the crystal structure of each homodimer, is shown in yellow and pY in red in the ball-stick representation. Right: overlay of the pY peptide linker and YHO-1701 in the SH2 domain of STAT1 generated by MOE. YHO-1701, shown in cyan, is located in the space-filling representation. b Human lung embryonic fibroblast cell lines (MRC-5 and IMR-90) were treated with YHO-1701 at stepwise concentrations for 72 h. Cell viability was quantified by MTT assay. Mean ± SD of experimental replicates (n = 6, representing separate wells) are shown. c H3122, H2228 and A925L cells were treated with alectinib (1 µM) or DMSO with or without YHO-1701 (3 µM) for 48 h. Cytoplasmic and nuclear fractions of the lysates were analyzed by western blotting with the indicated antibodies. d H3122, H2228, and A925L cells were treated with ALK-TKIs (alectinib, ceritinib, lorlatinib, and brigatinib: 1 µM) or DMSO with or without YHO-1701 (3 µM) for 48 h. The lysates were analyzed by western blotting with the indicated antibodies.

## Slide 6
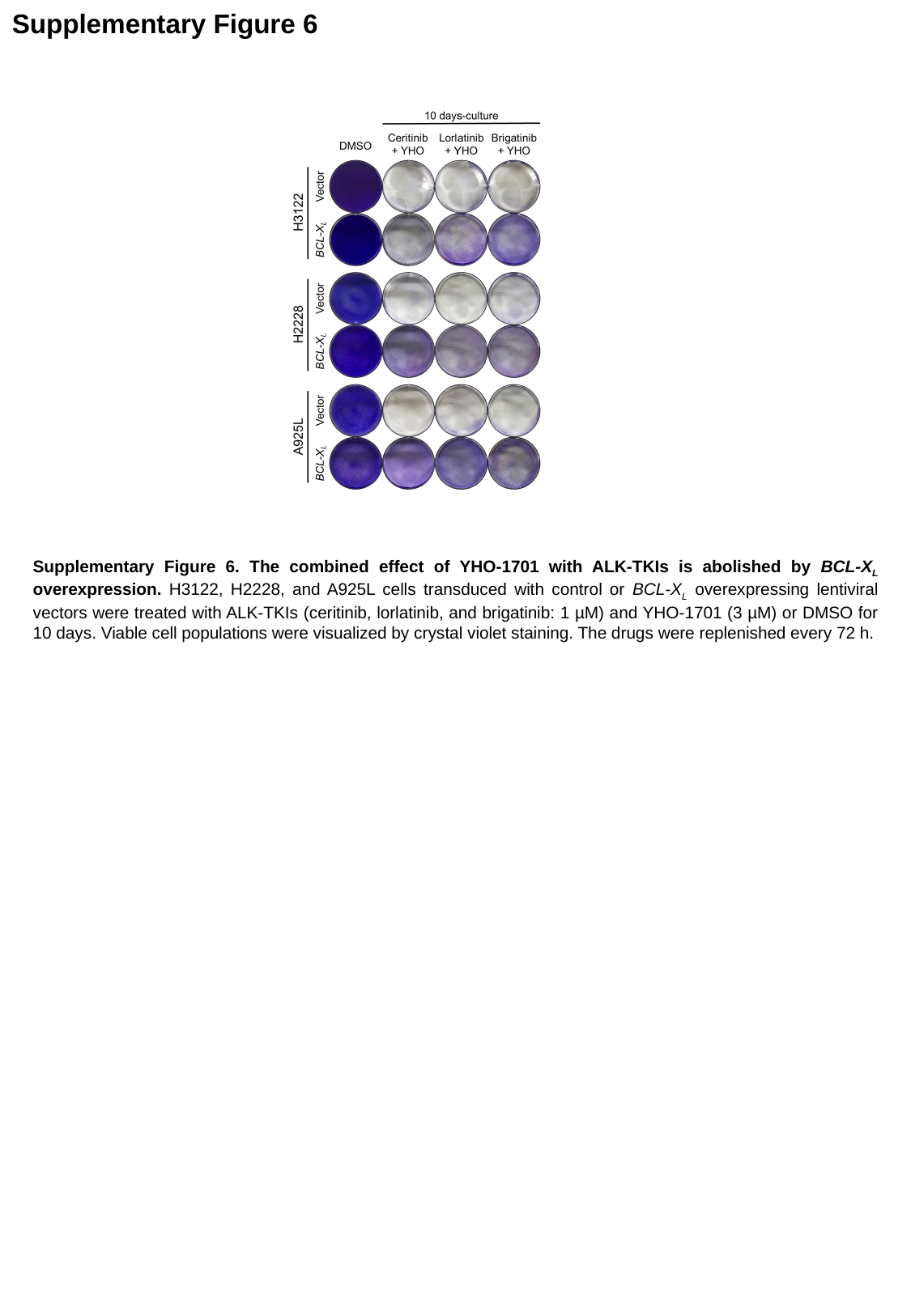

Supplementary Figure 6
Supplementary Figure 6. The combined effect of YHO-1701 with ALK-TKIs is abolished by BCL-XL overexpression. H3122, H2228, and A925L cells transduced with control or BCL-XL overexpressing lentiviral vectors were treated with ALK-TKIs (ceritinib, lorlatinib, and brigatinib: 1 µM) and YHO-1701 (3 µM) or DMSO for 10 days. Viable cell populations were visualized by crystal violet staining. The drugs were replenished every 72 h.

## Slide 7
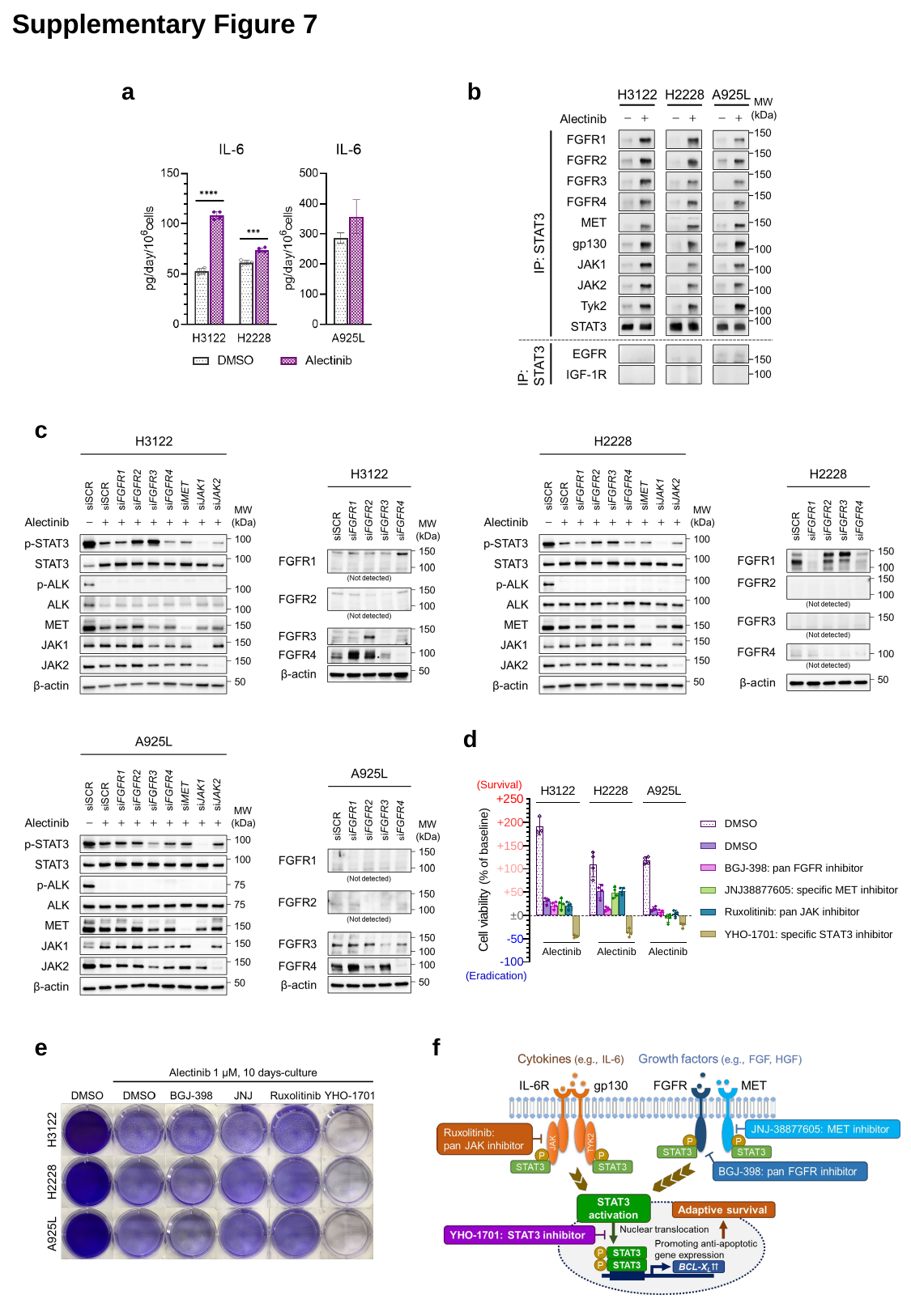

Supplementary Figure 7
a
b
c
d
e
f

## Slide 8
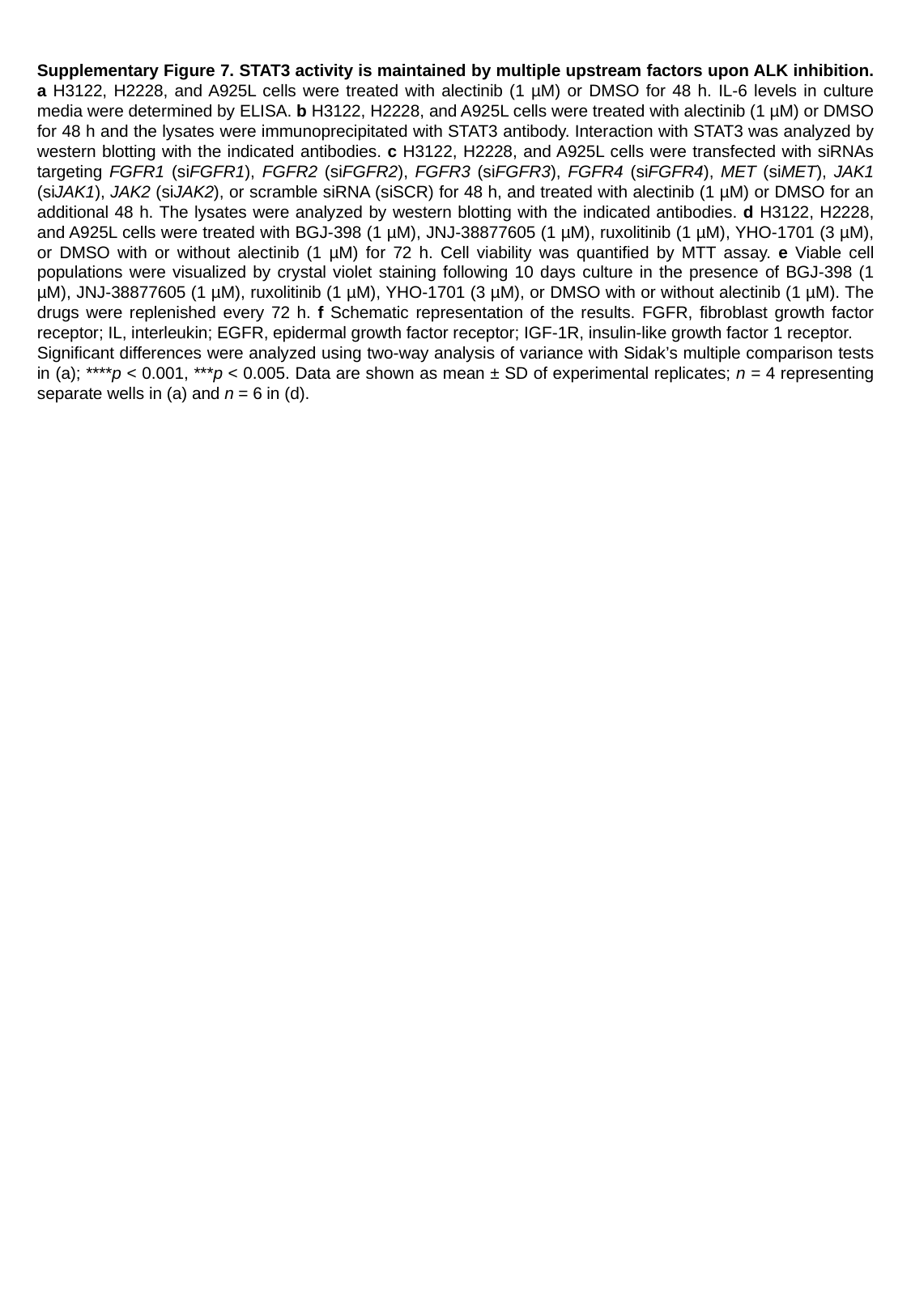

Supplementary Figure 7. STAT3 activity is maintained by multiple upstream factors upon ALK inhibition. a H3122, H2228, and A925L cells were treated with alectinib (1 µM) or DMSO for 48 h. IL-6 levels in culture media were determined by ELISA. b H3122, H2228, and A925L cells were treated with alectinib (1 µM) or DMSO for 48 h and the lysates were immunoprecipitated with STAT3 antibody. Interaction with STAT3 was analyzed by western blotting with the indicated antibodies. c H3122, H2228, and A925L cells were transfected with siRNAs targeting FGFR1 (siFGFR1), FGFR2 (siFGFR2), FGFR3 (siFGFR3), FGFR4 (siFGFR4), MET (siMET), JAK1 (siJAK1), JAK2 (siJAK2), or scramble siRNA (siSCR) for 48 h, and treated with alectinib (1 µM) or DMSO for an additional 48 h. The lysates were analyzed by western blotting with the indicated antibodies. d H3122, H2228, and A925L cells were treated with BGJ-398 (1 µM), JNJ-38877605 (1 µM), ruxolitinib (1 µM), YHO-1701 (3 µM), or DMSO with or without alectinib (1 µM) for 72 h. Cell viability was quantified by MTT assay. e Viable cell populations were visualized by crystal violet staining following 10 days culture in the presence of BGJ-398 (1 µM), JNJ-38877605 (1 µM), ruxolitinib (1 µM), YHO-1701 (3 µM), or DMSO with or without alectinib (1 µM). The drugs were replenished every 72 h. f Schematic representation of the results. FGFR, fibroblast growth factor receptor; IL, interleukin; EGFR, epidermal growth factor receptor; IGF-1R, insulin-like growth factor 1 receptor.
Significant differences were analyzed using two-way analysis of variance with Sidak’s multiple comparison tests in (a); ****p < 0.001, ***p < 0.005. Data are shown as mean ± SD of experimental replicates; n = 4 representing separate wells in (a) and n = 6 in (d).

## Slide 9
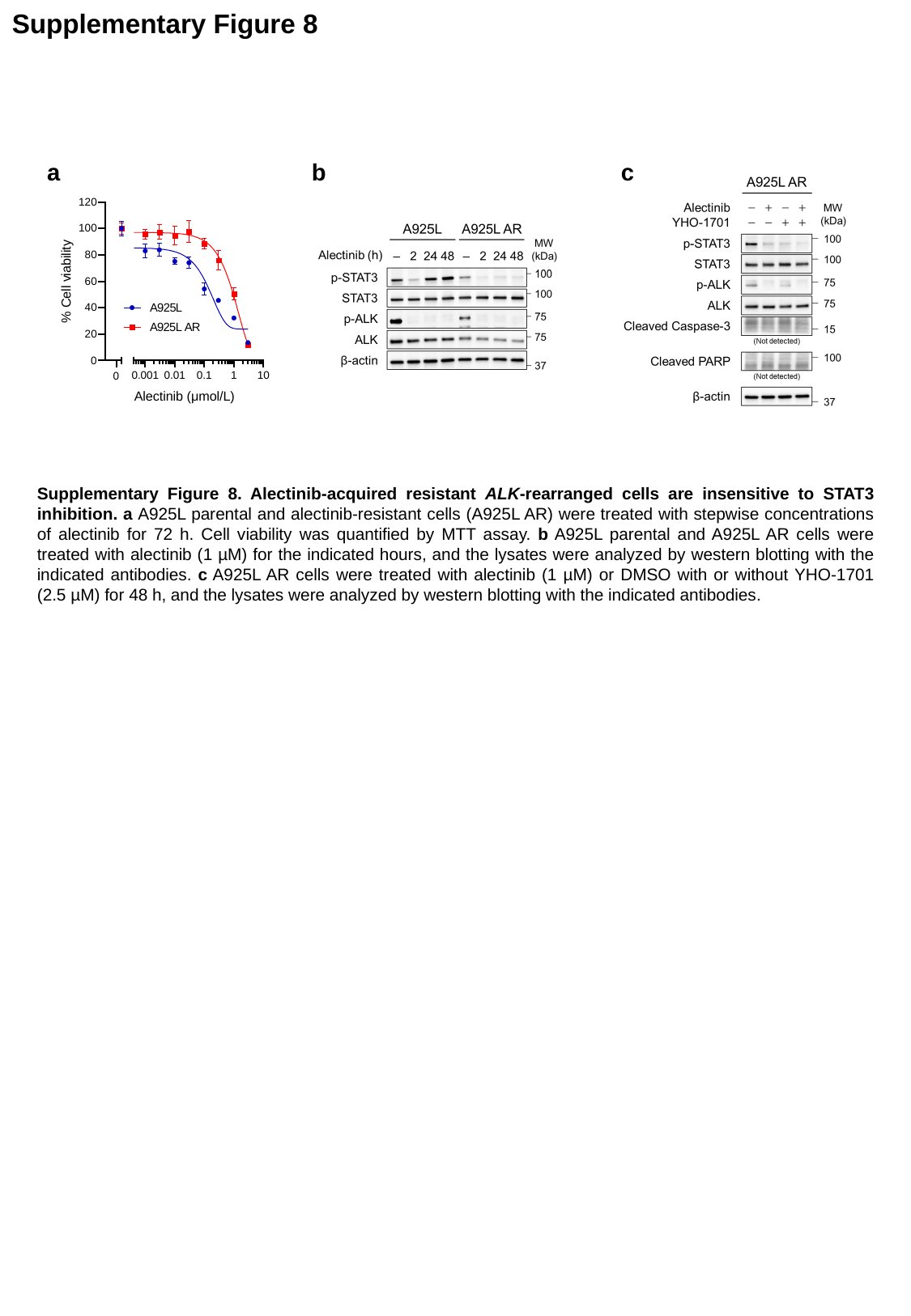

Supplementary Figure 8
c
a
b
Supplementary Figure 8. Alectinib-acquired resistant ALK-rearranged cells are insensitive to STAT3 inhibition. a A925L parental and alectinib-resistant cells (A925L AR) were treated with stepwise concentrations of alectinib for 72 h. Cell viability was quantified by MTT assay. b A925L parental and A925L AR cells were treated with alectinib (1 µM) for the indicated hours, and the lysates were analyzed by western blotting with the indicated antibodies. c A925L AR cells were treated with alectinib (1 µM) or DMSO with or without YHO-1701 (2.5 µM) for 48 h, and the lysates were analyzed by western blotting with the indicated antibodies.

## Slide 10
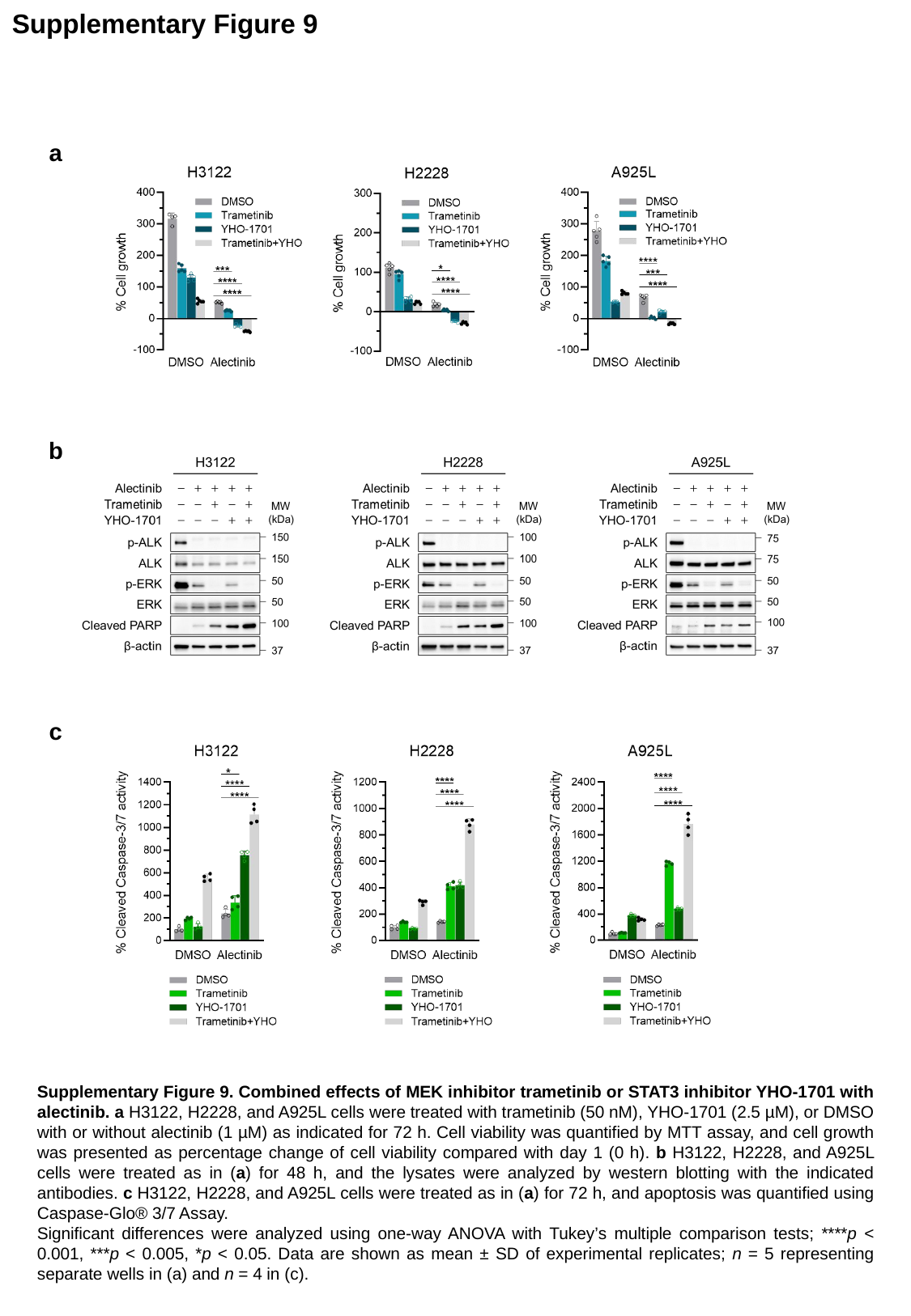

Supplementary Figure 9
a
b
c
Supplementary Figure 9. Combined effects of MEK inhibitor trametinib or STAT3 inhibitor YHO-1701 with alectinib. a H3122, H2228, and A925L cells were treated with trametinib (50 nM), YHO-1701 (2.5 µM), or DMSO with or without alectinib (1 µM) as indicated for 72 h. Cell viability was quantified by MTT assay, and cell growth was presented as percentage change of cell viability compared with day 1 (0 h). b H3122, H2228, and A925L cells were treated as in (a) for 48 h, and the lysates were analyzed by western blotting with the indicated antibodies. c H3122, H2228, and A925L cells were treated as in (a) for 72 h, and apoptosis was quantified using Caspase-Glo® 3/7 Assay.
Significant differences were analyzed using one-way ANOVA with Tukey’s multiple comparison tests; ****p < 0.001, ***p < 0.005, *p < 0.05. Data are shown as mean ± SD of experimental replicates; n = 5 representing separate wells in (a) and n = 4 in (c).

## Slide 11
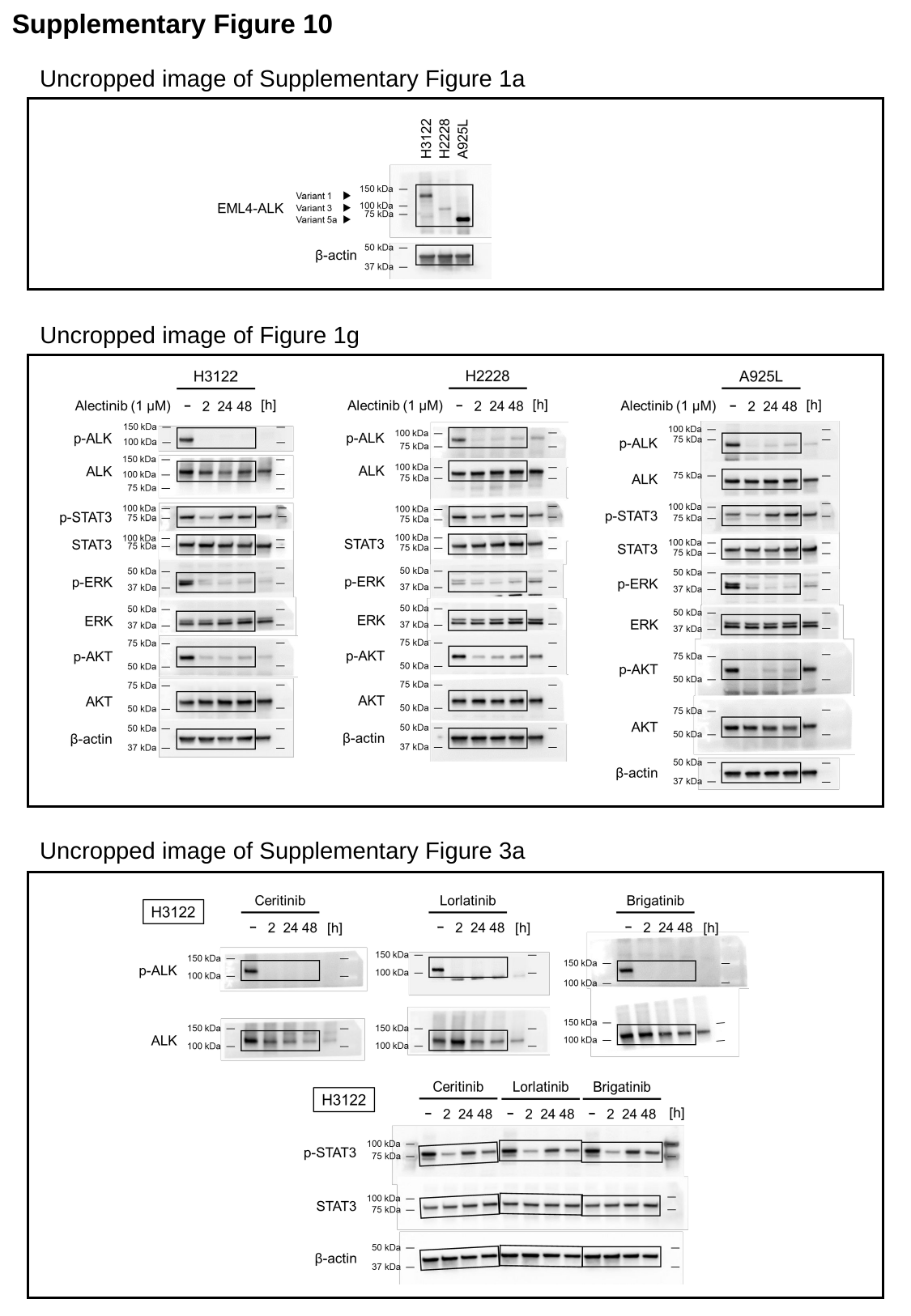

Supplementary Figure 10
Uncropped image of Supplementary Figure 1a
Uncropped image of Figure 1g
Uncropped image of Supplementary Figure 3a

## Slide 12
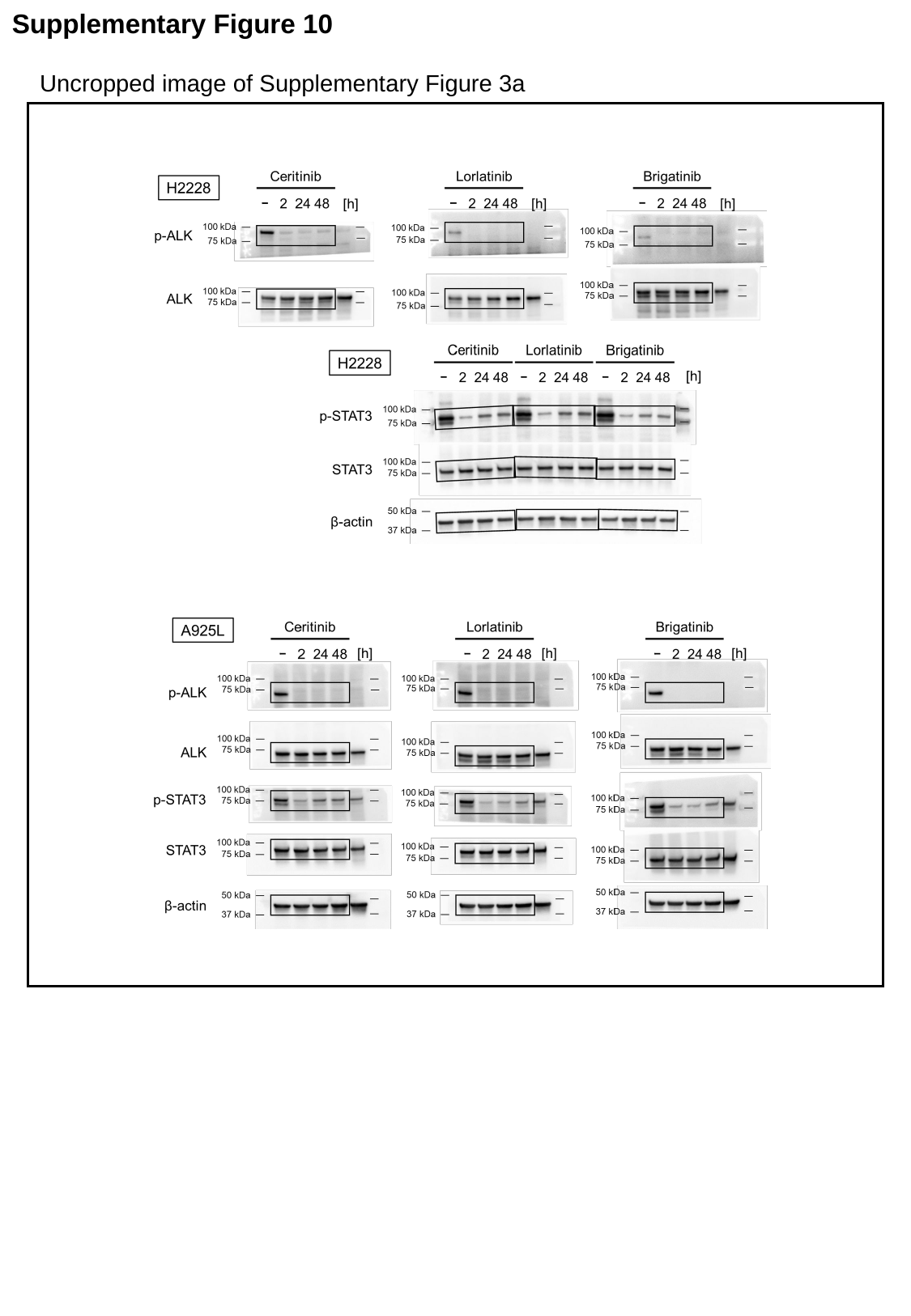

Supplementary Figure 10
Uncropped image of Supplementary Figure 3a

## Slide 13
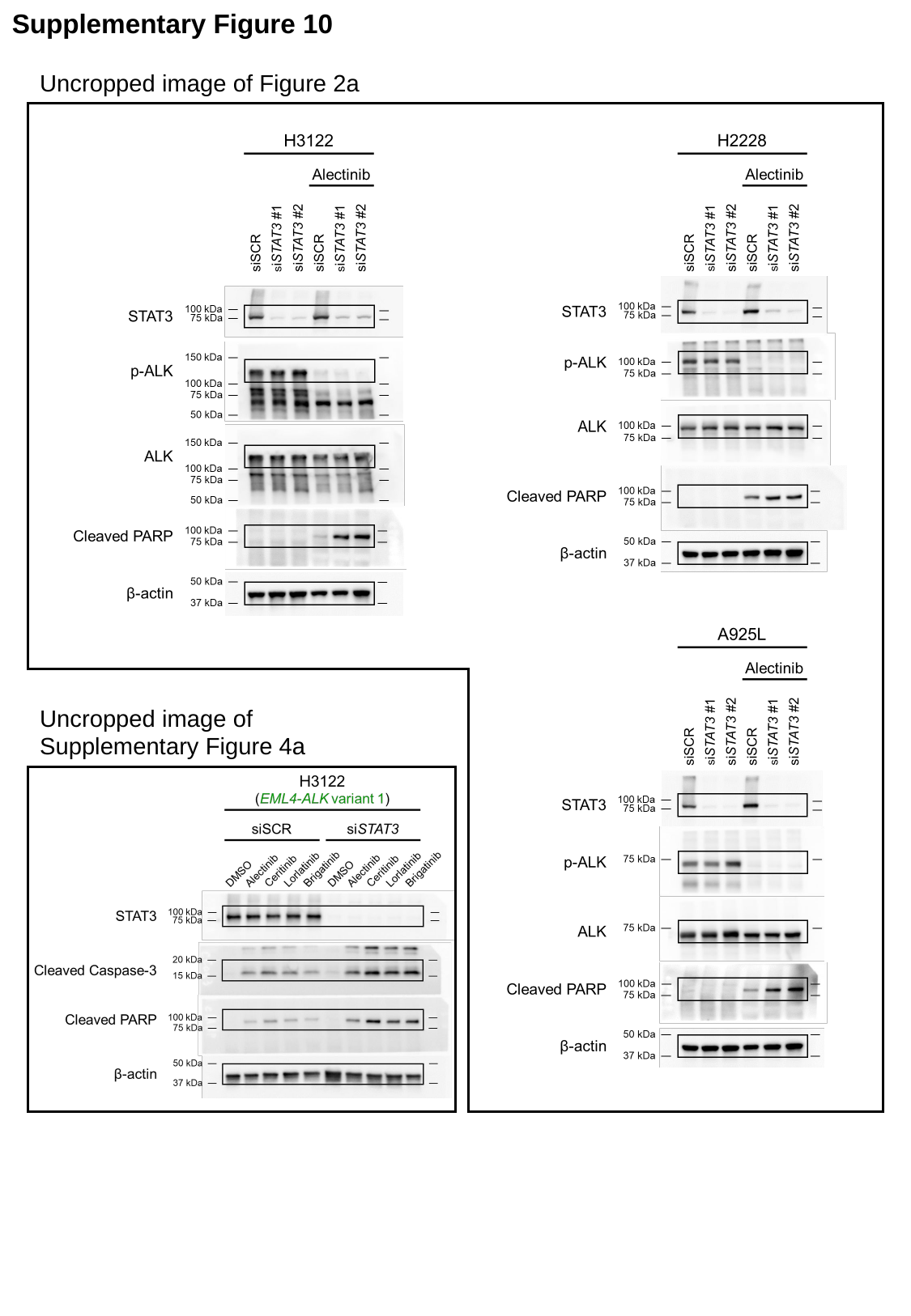

Supplementary Figure 10
Uncropped image of Figure 2a
Uncropped image of
Supplementary Figure 4a

## Slide 14
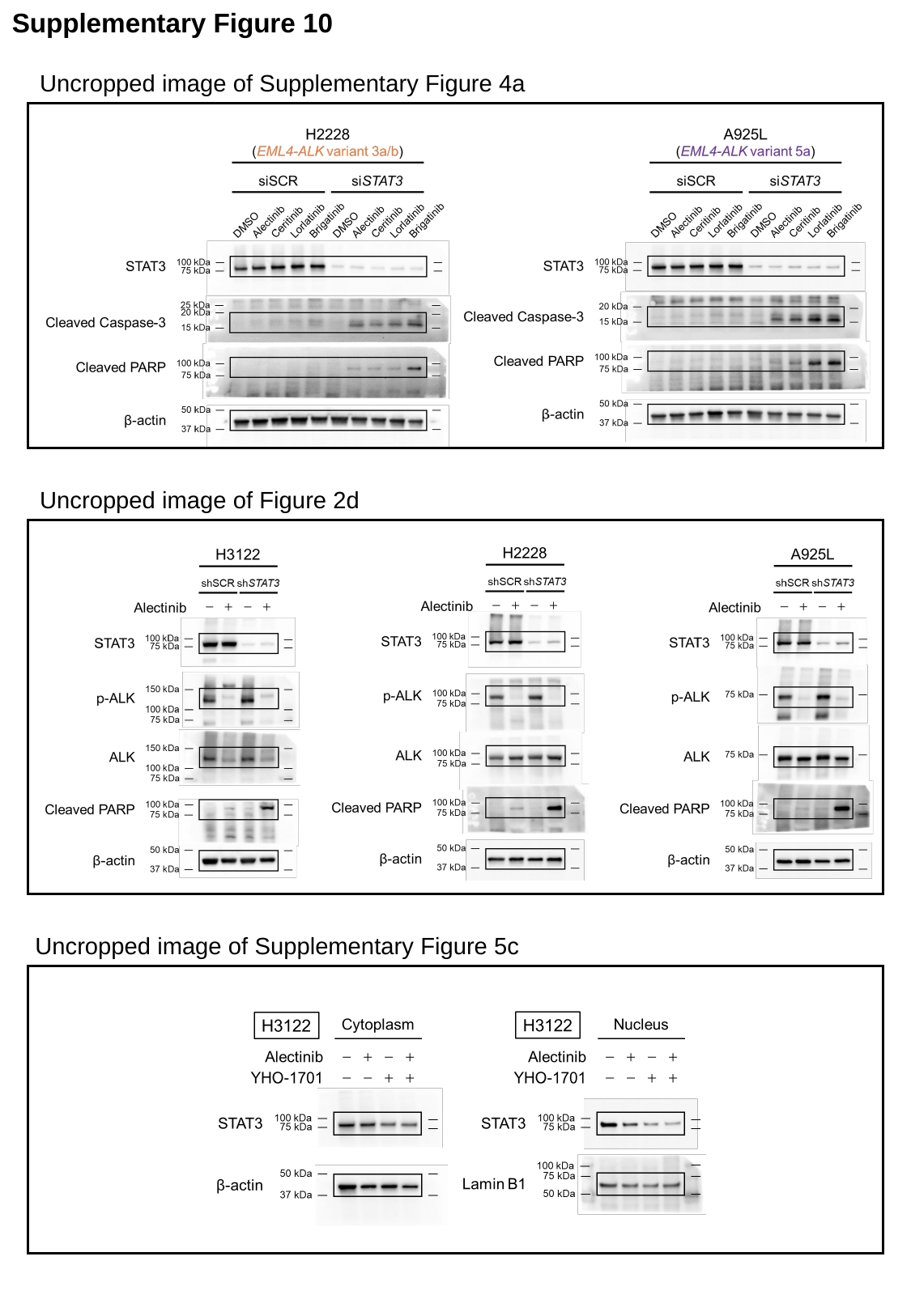

Supplementary Figure 10
Uncropped image of Supplementary Figure 4a
Uncropped image of Figure 2d
Uncropped image of Supplementary Figure 5c

## Slide 15
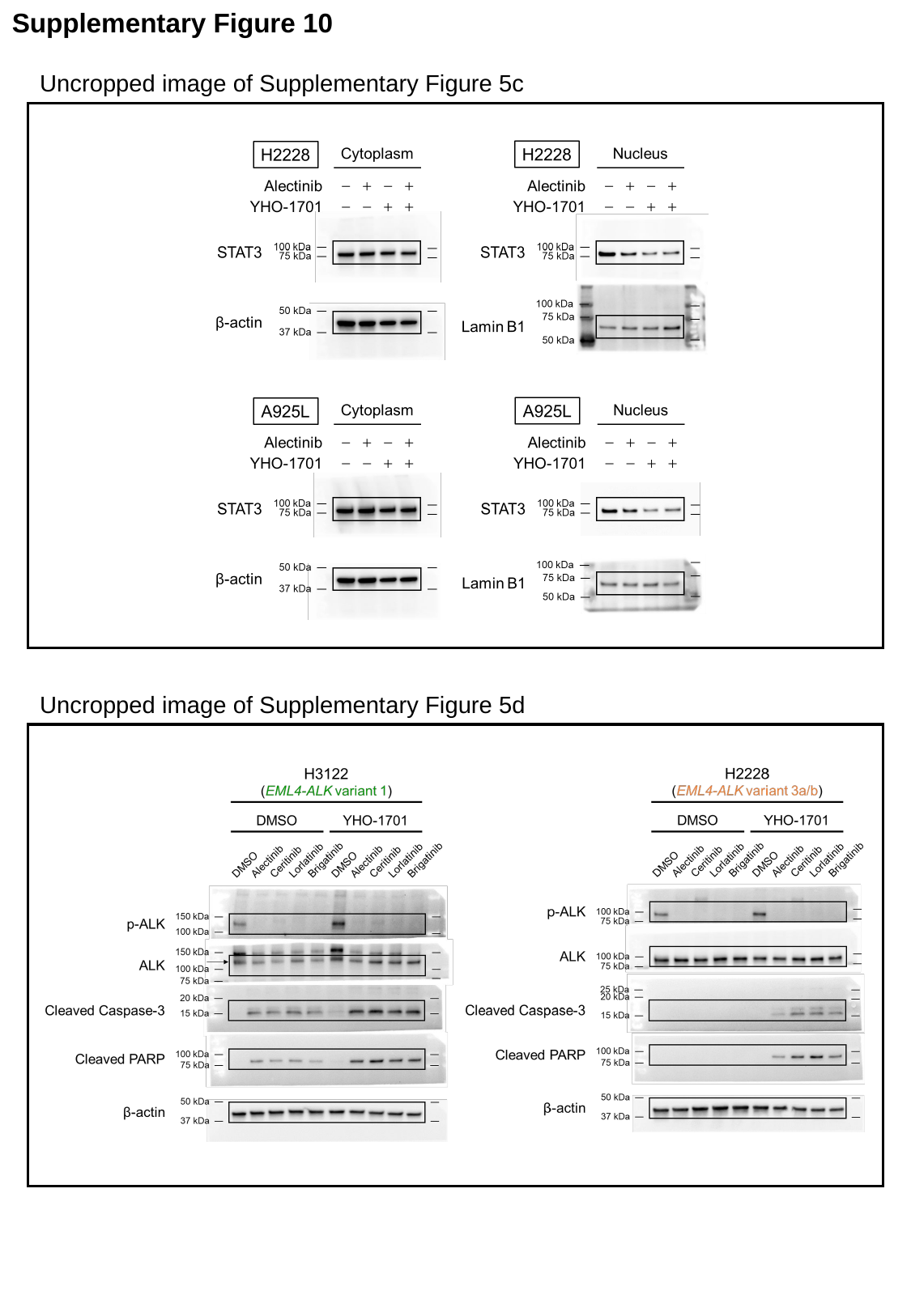

Supplementary Figure 10
Uncropped image of Supplementary Figure 5c
Uncropped image of Supplementary Figure 5d

## Slide 16
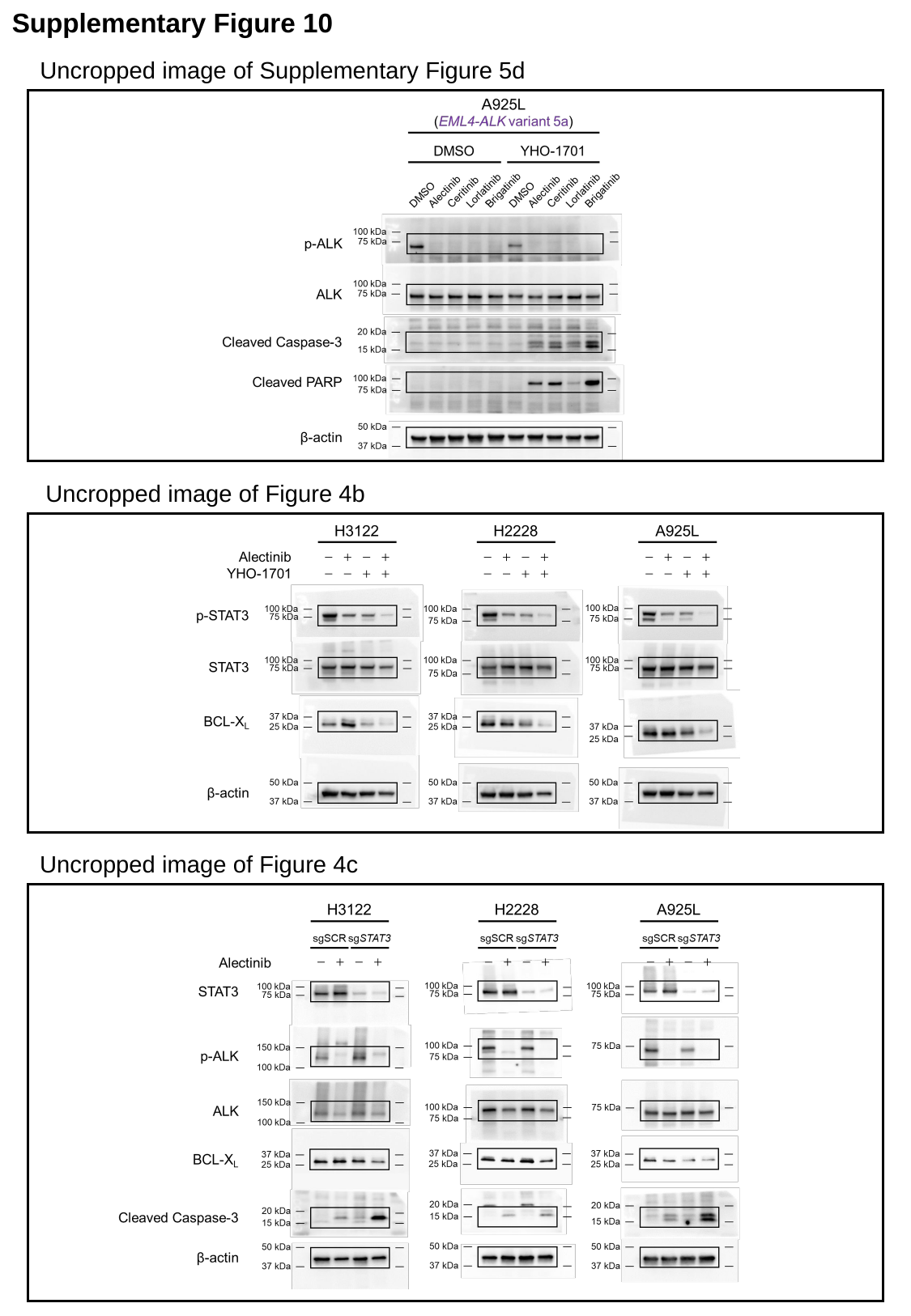

Supplementary Figure 10
Uncropped image of Supplementary Figure 5d
Uncropped image of Figure 4b
Uncropped image of Figure 4c

## Slide 17
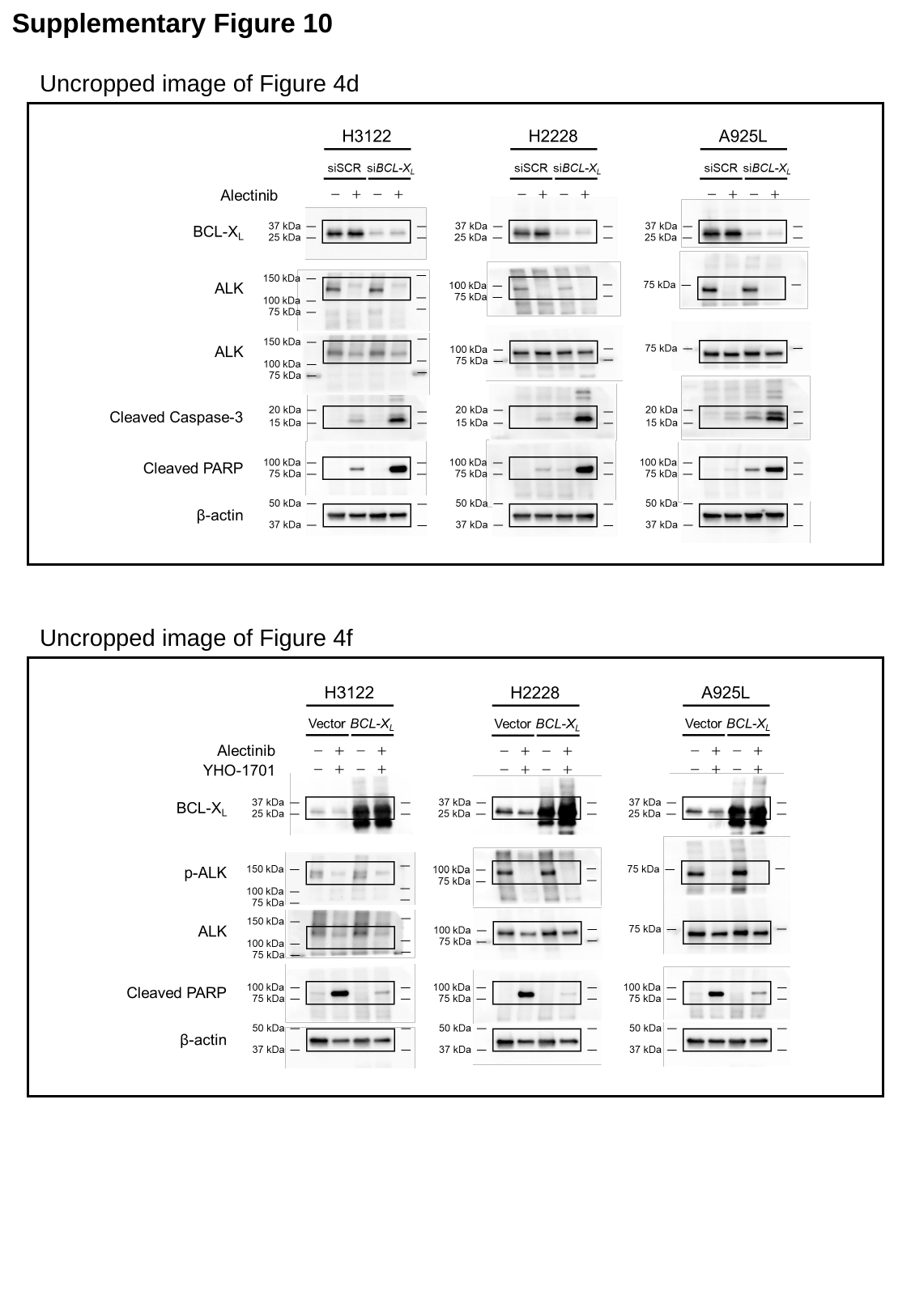

Supplementary Figure 10
Uncropped image of Figure 4d
Uncropped image of Figure 4f

## Slide 18
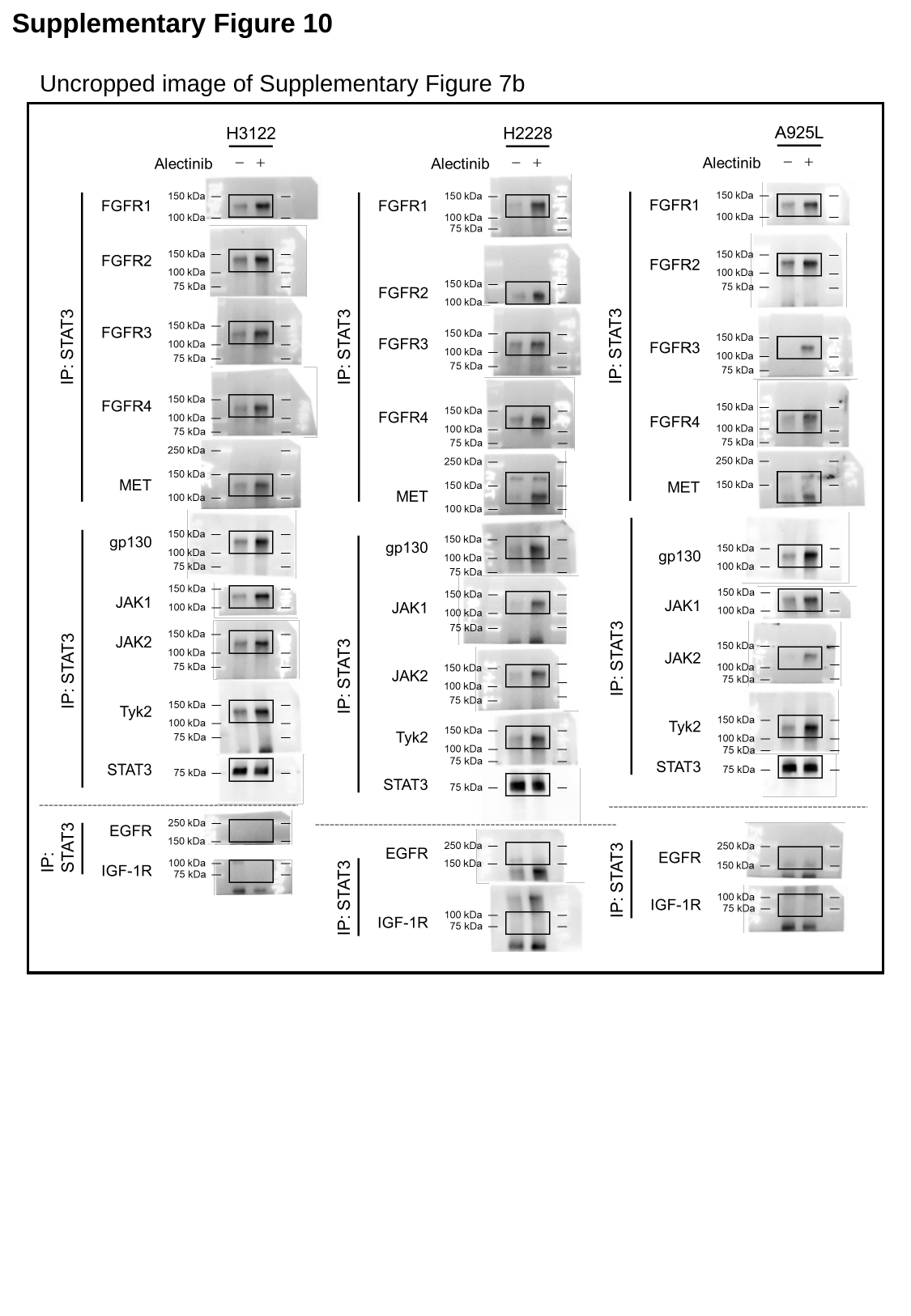

Supplementary Figure 10
Uncropped image of Supplementary Figure 7b

## Slide 19
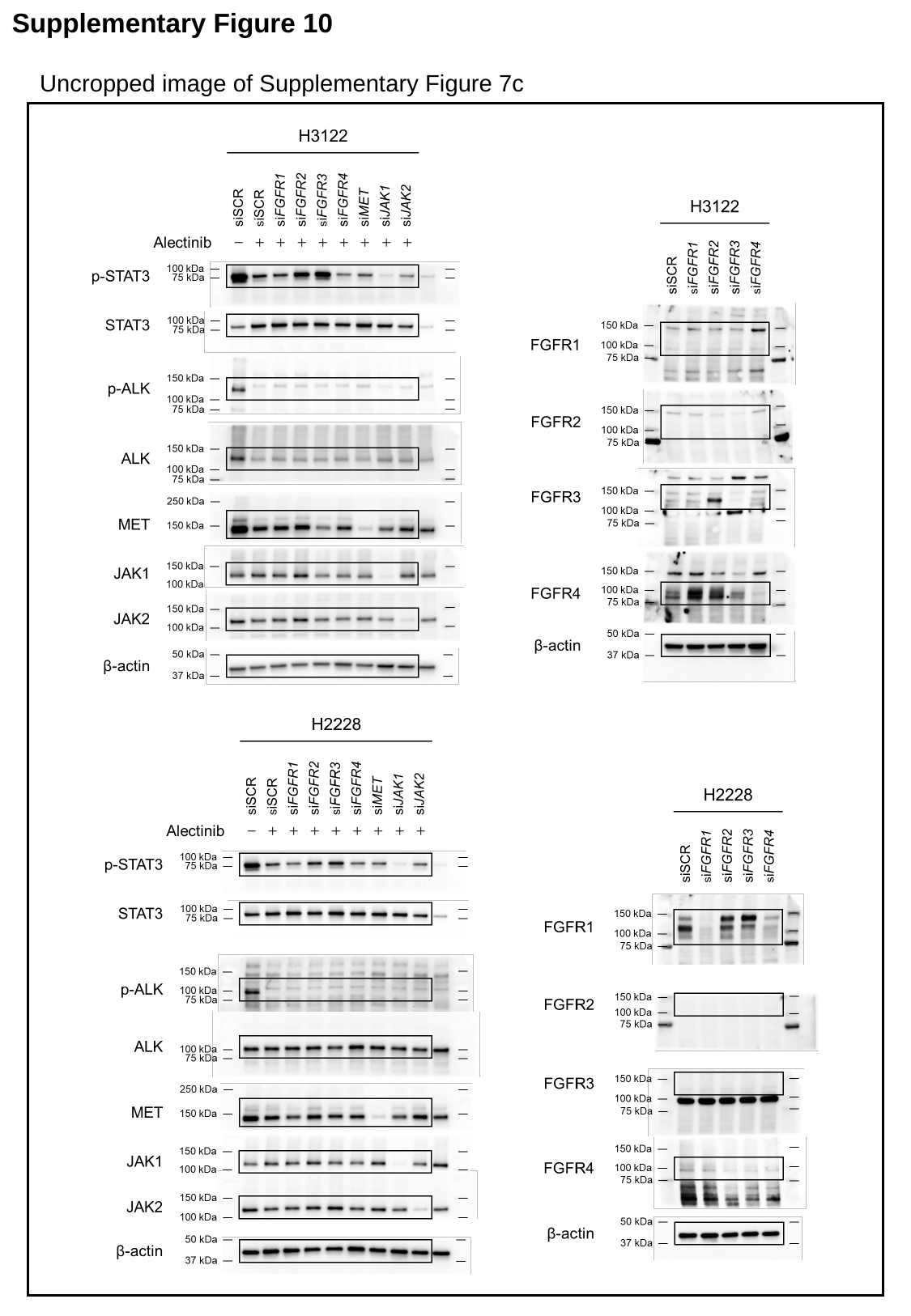

Supplementary Figure 10
Uncropped image of Supplementary Figure 7c

## Slide 20
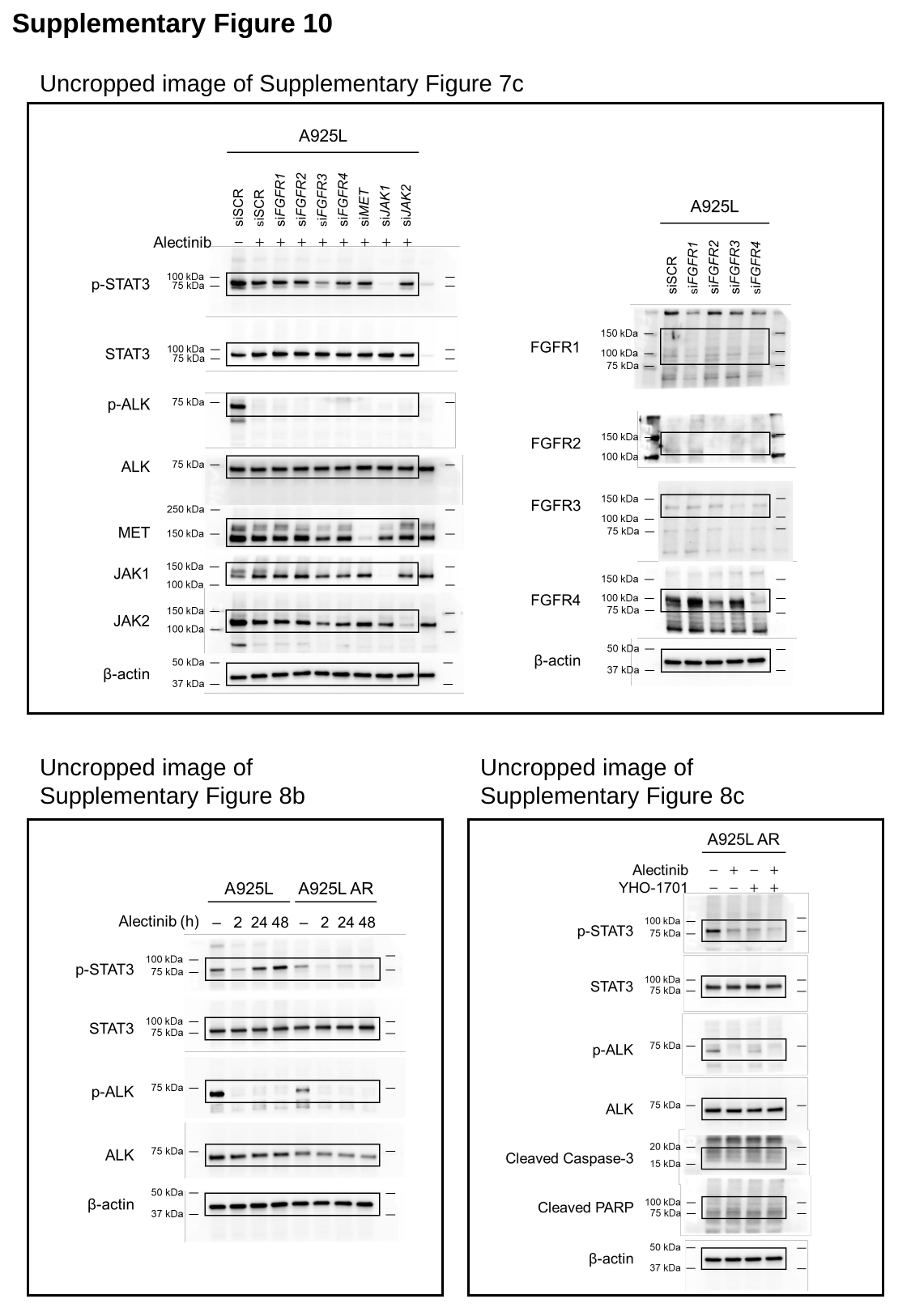

Supplementary Figure 10
Uncropped image of Supplementary Figure 7c
Uncropped image of
Supplementary Figure 8b
Uncropped image of
Supplementary Figure 8c

## Slide 21
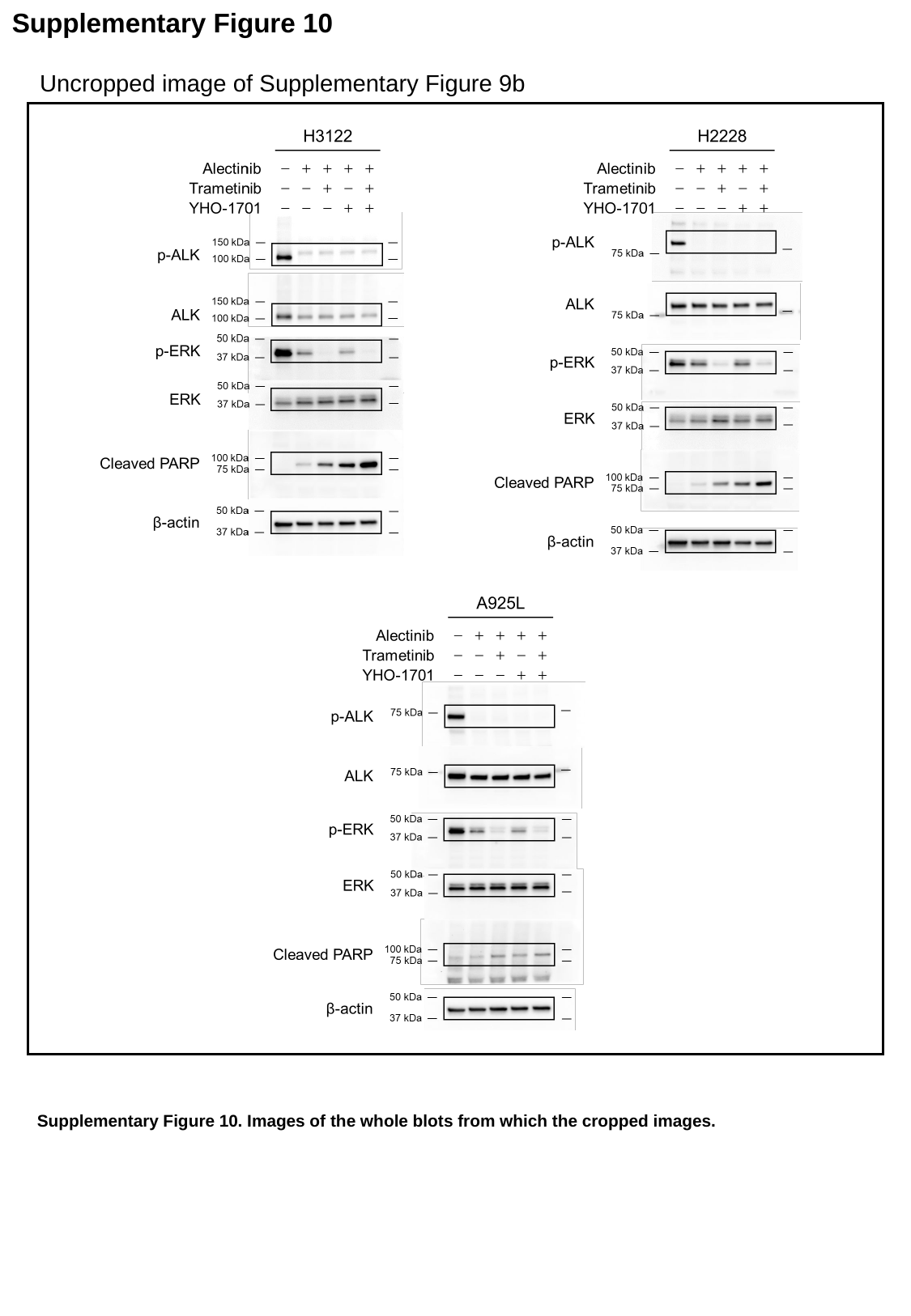

Supplementary Figure 10
Uncropped image of Supplementary Figure 9b
Supplementary Figure 10. Images of the whole blots from which the cropped images.

## Slide 22
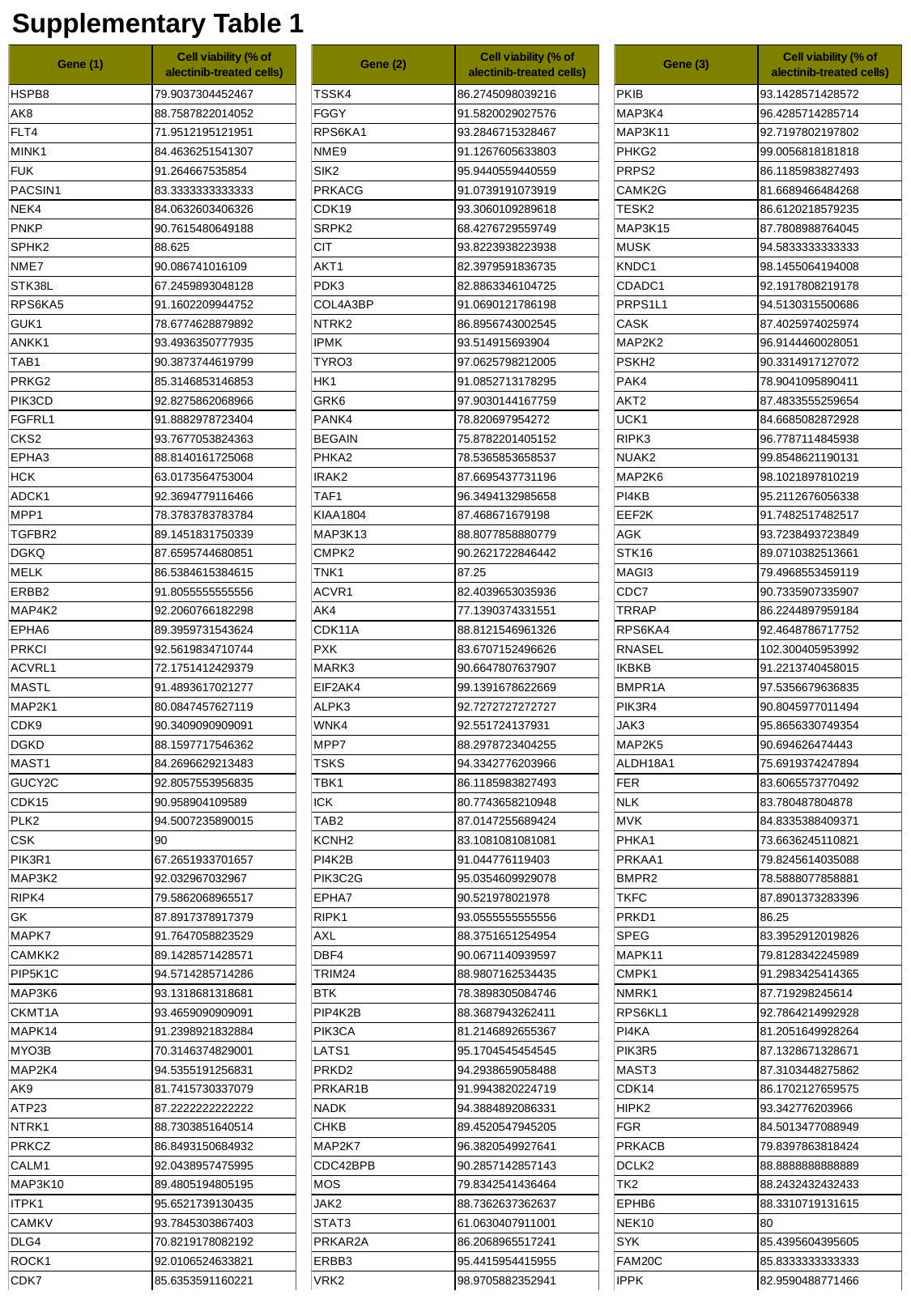

Supplementary Table 1

## Slide 23
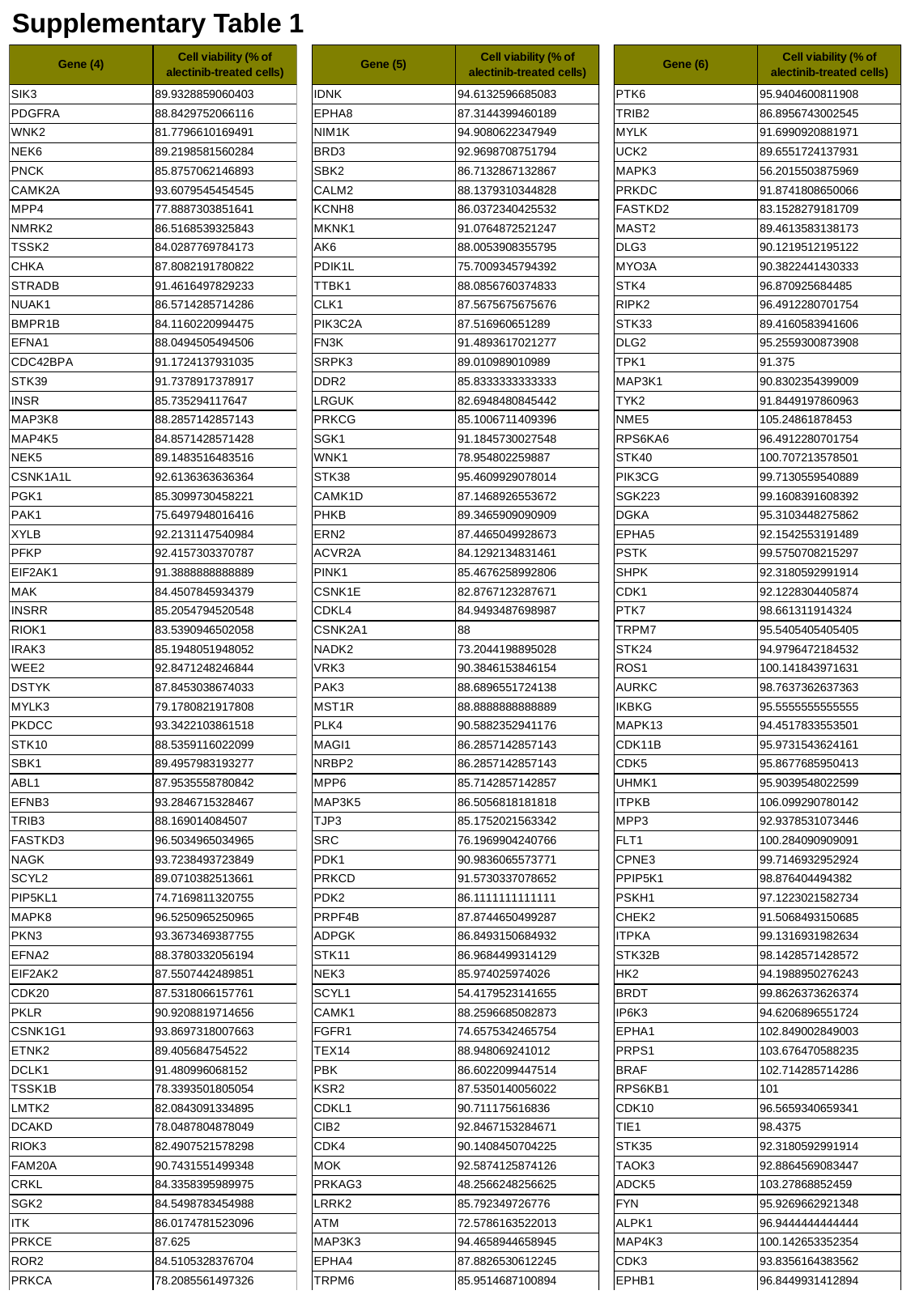

Supplementary Table 1

## Slide 24
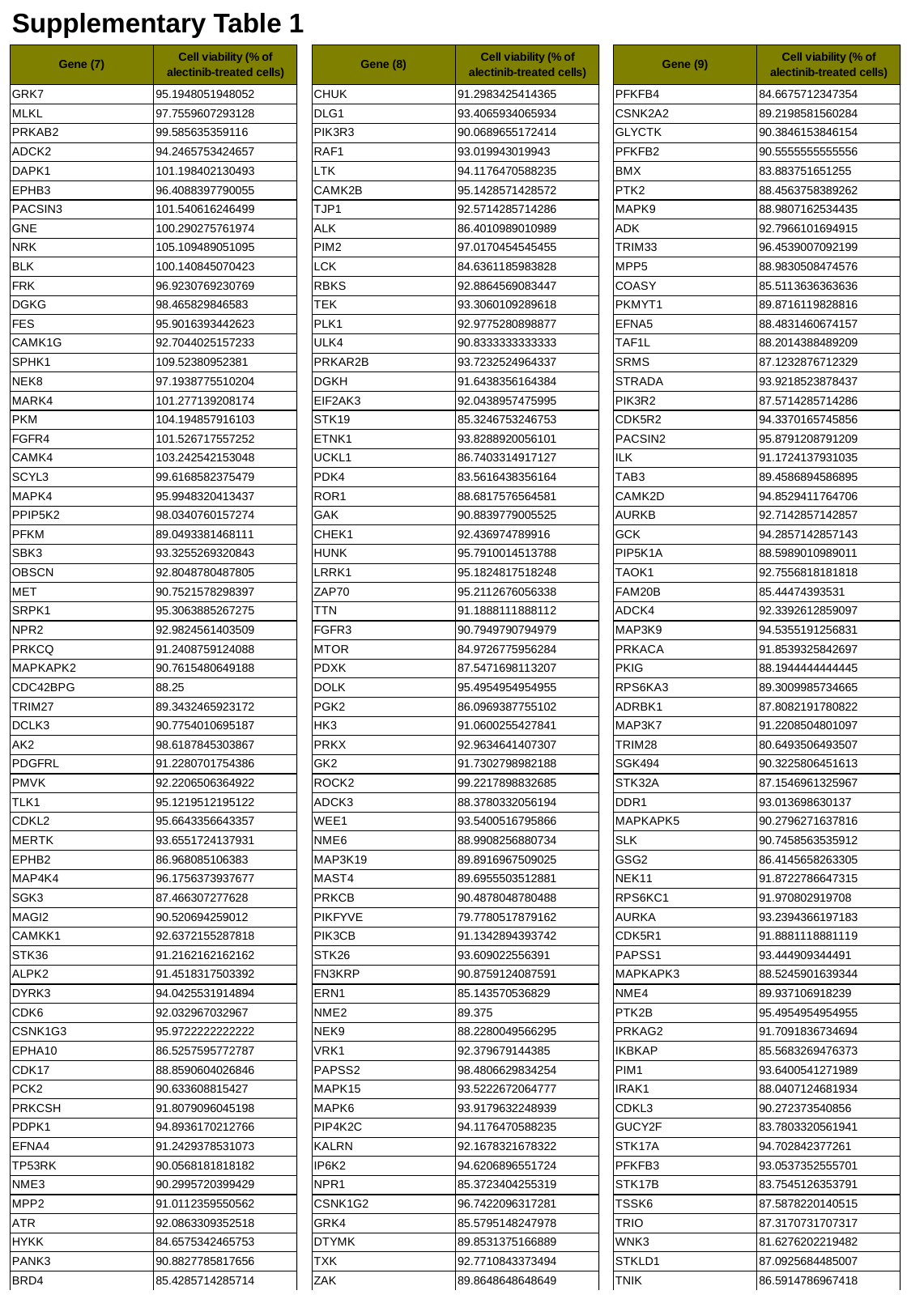

Supplementary Table 1

## Slide 25
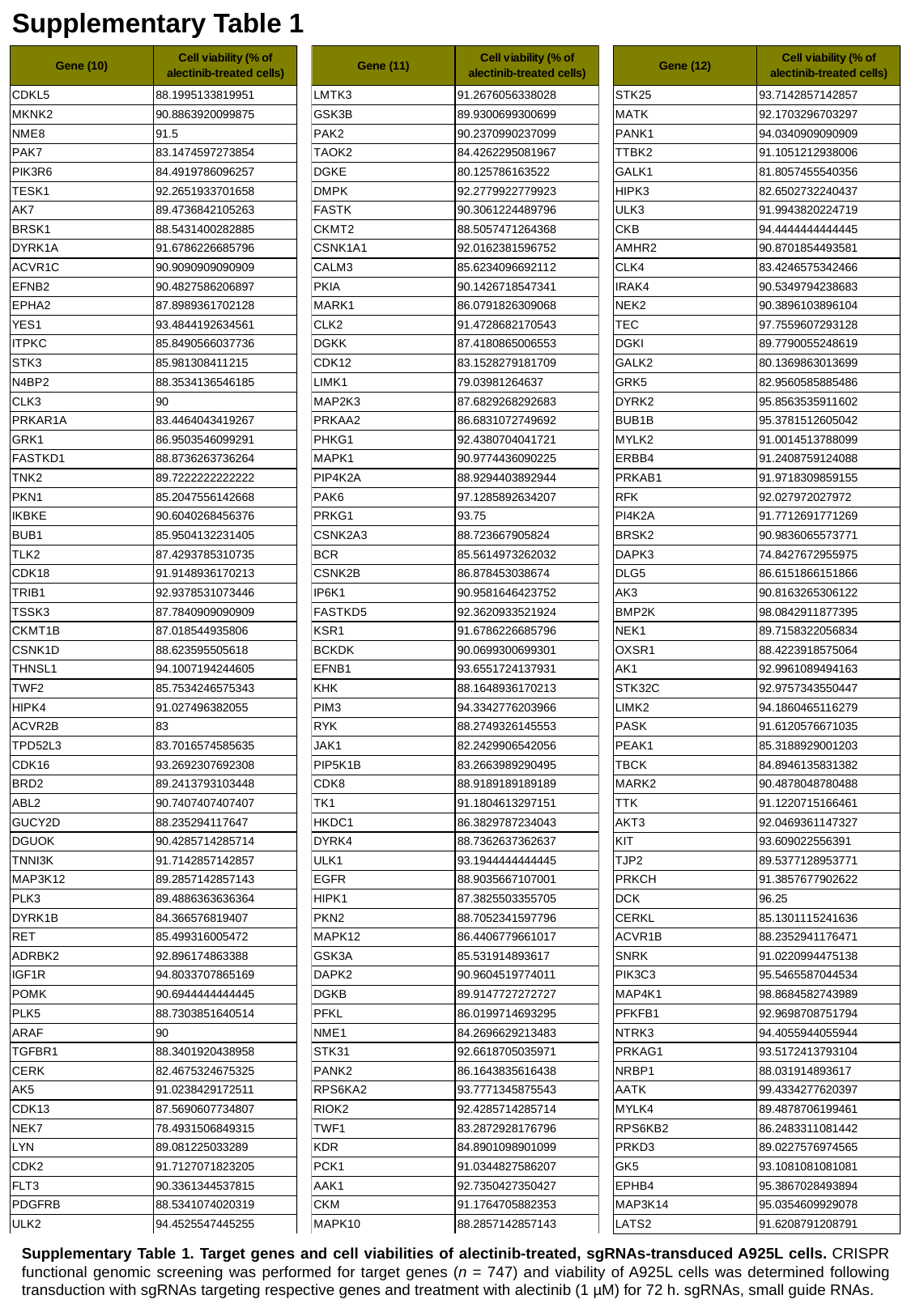

Supplementary Table 1
Supplementary Table 1. Target genes and cell viabilities of alectinib-treated, sgRNAs-transduced A925L cells. CRISPR functional genomic screening was performed for target genes (n = 747) and viability of A925L cells was determined following transduction with sgRNAs targeting respective genes and treatment with alectinib (1 µM) for 72 h. sgRNAs, small guide RNAs.

## Slide 26
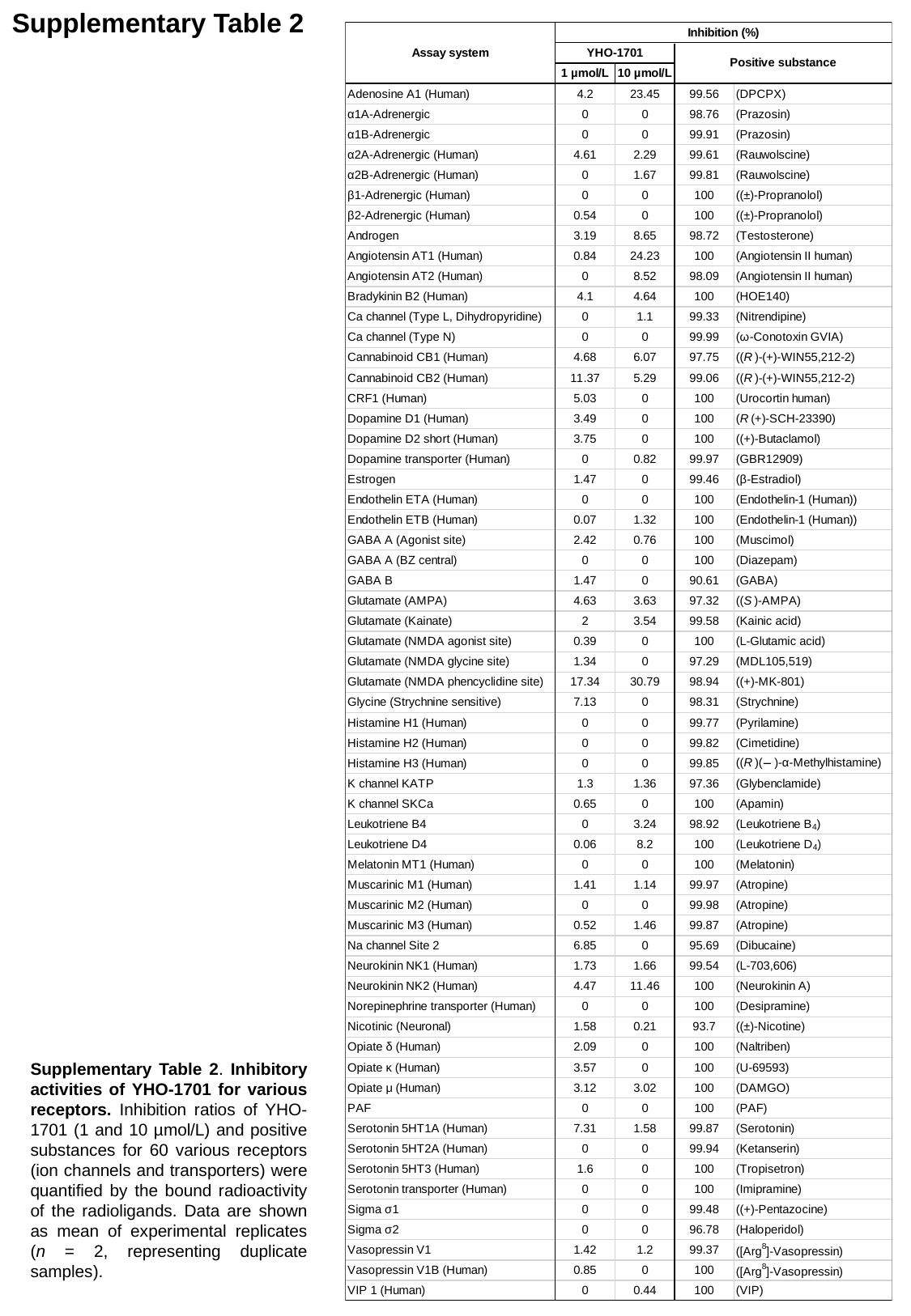

Supplementary Table 2
Supplementary Table 2. Inhibitory activities of YHO-1701 for various receptors. Inhibition ratios of YHO-1701 (1 and 10 µmol/L) and positive substances for 60 various receptors (ion channels and transporters) were quantified by the bound radioactivity of the radioligands. Data are shown as mean of experimental replicates (n = 2, representing duplicate samples).

## Slide 27
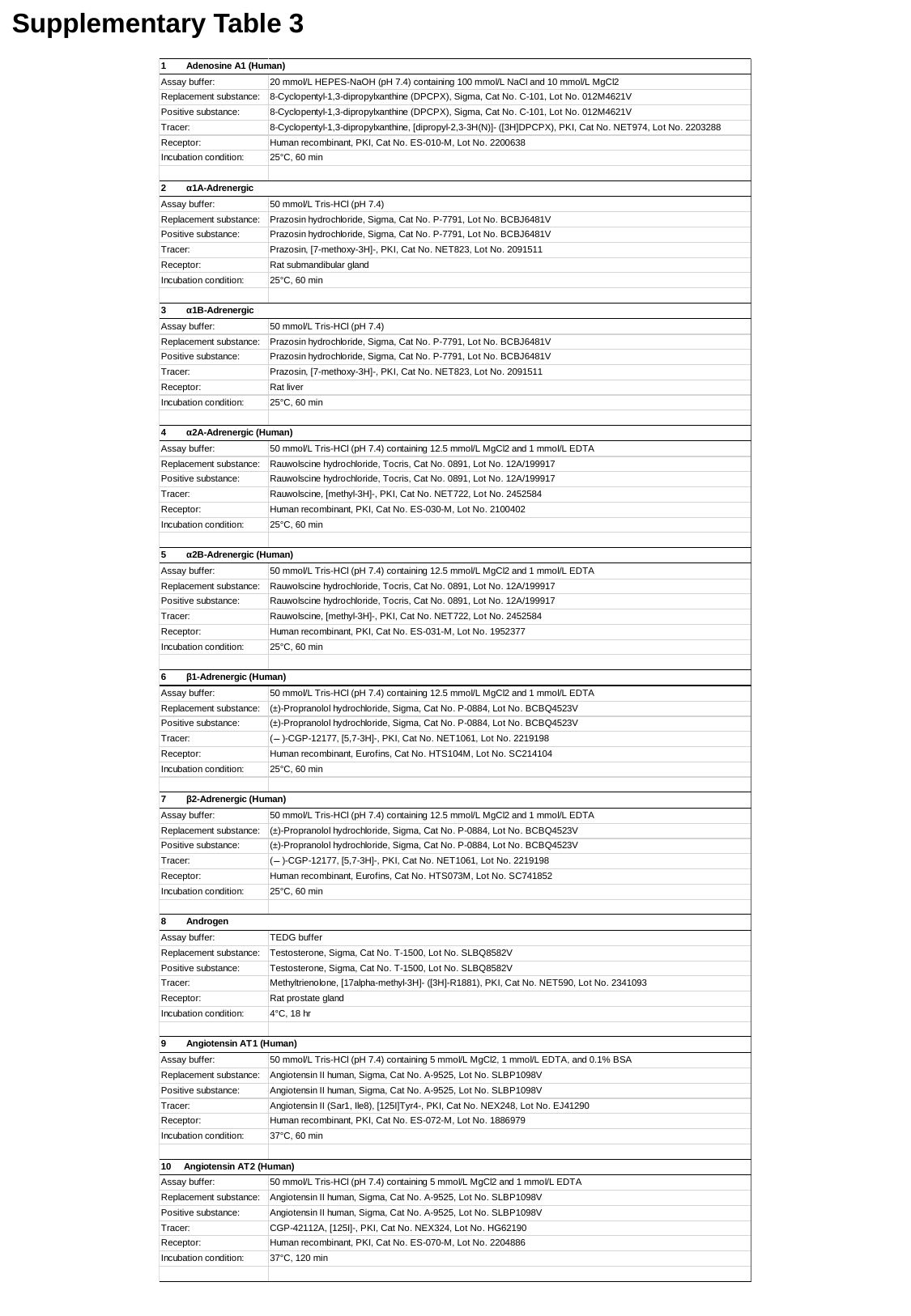

Supplementary Table 3

## Slide 28
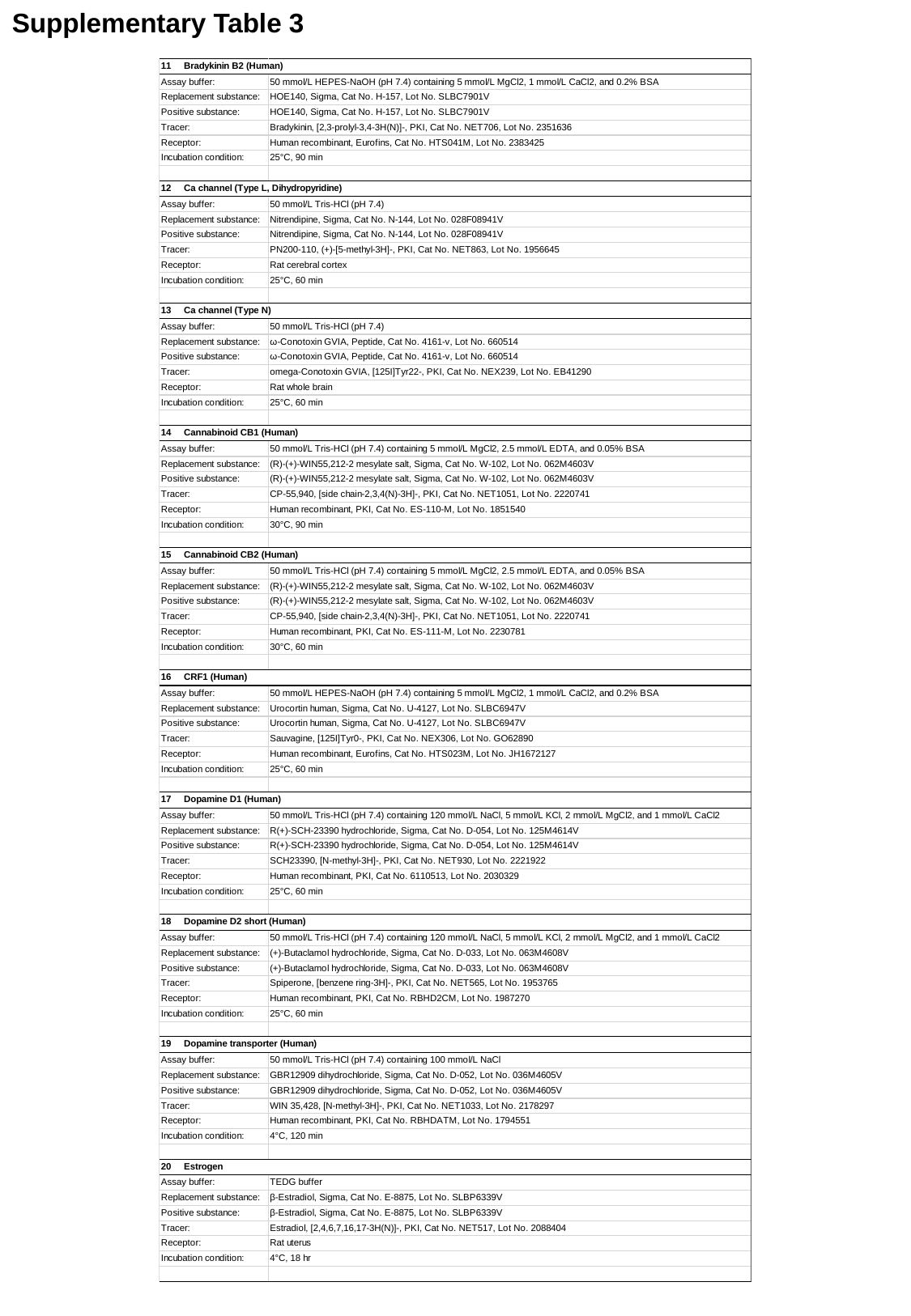

Supplementary Table 3

## Slide 29
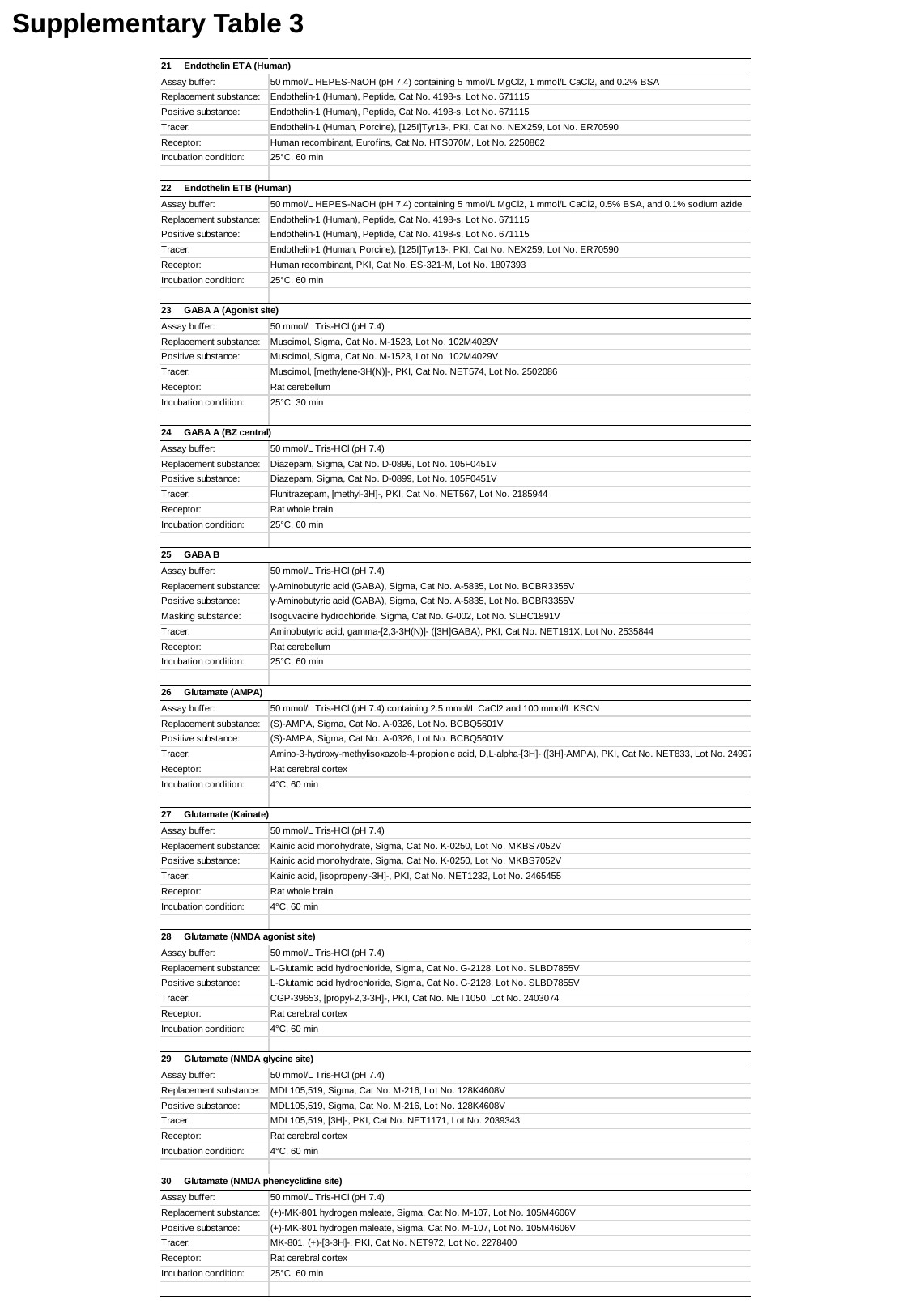

Supplementary Table 3

## Slide 30
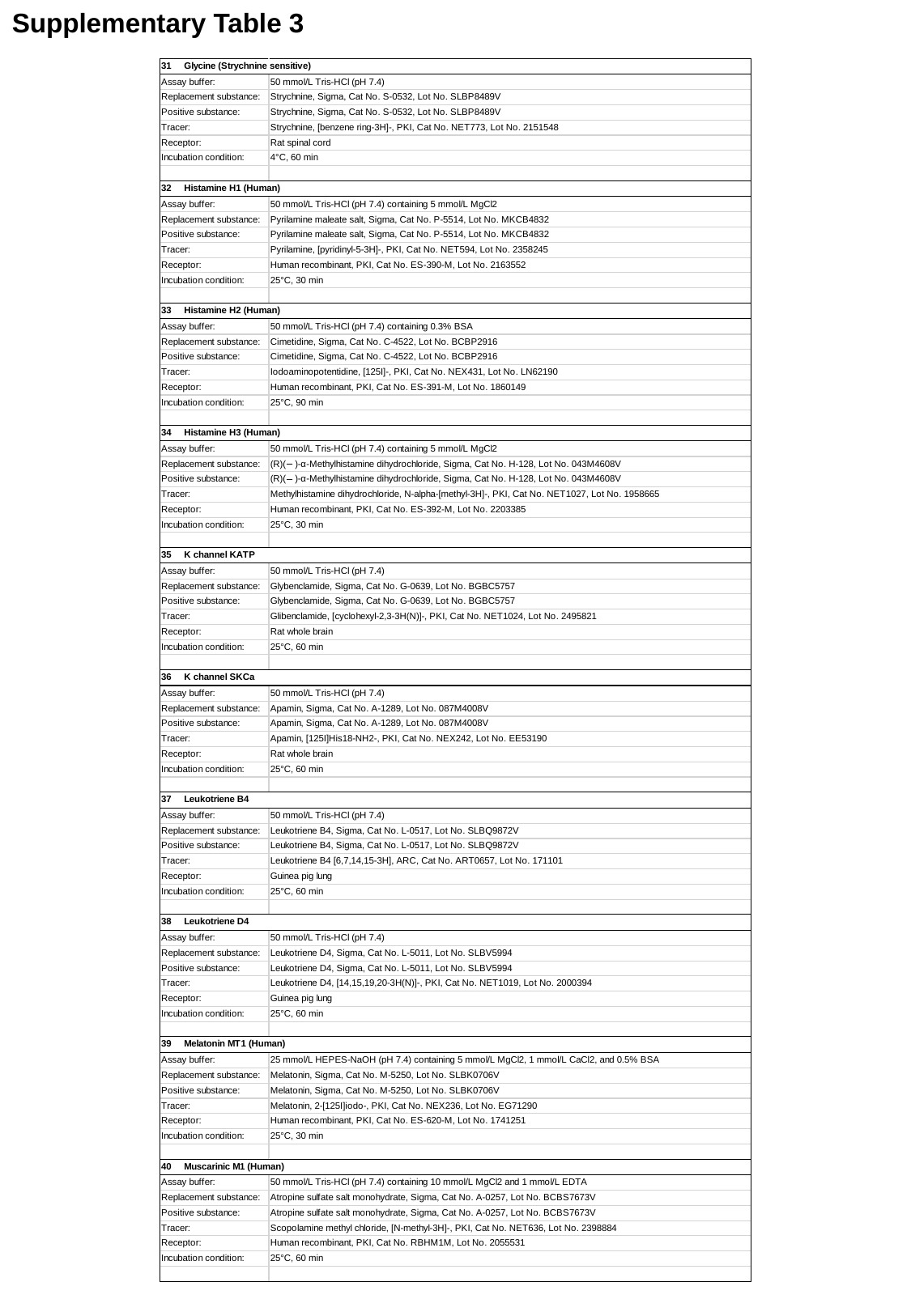

Supplementary Table 3

## Slide 31
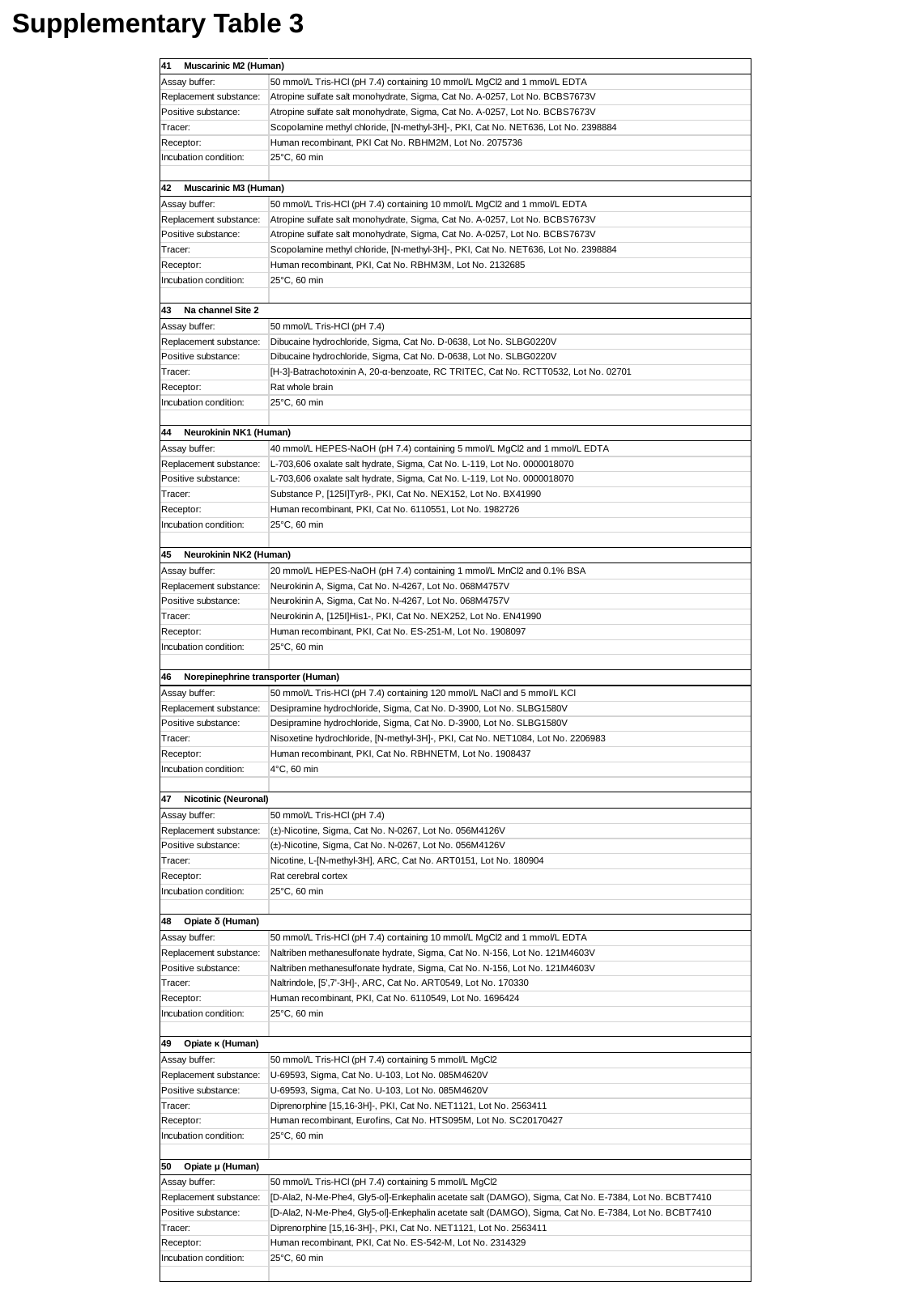

Supplementary Table 3

## Slide 32
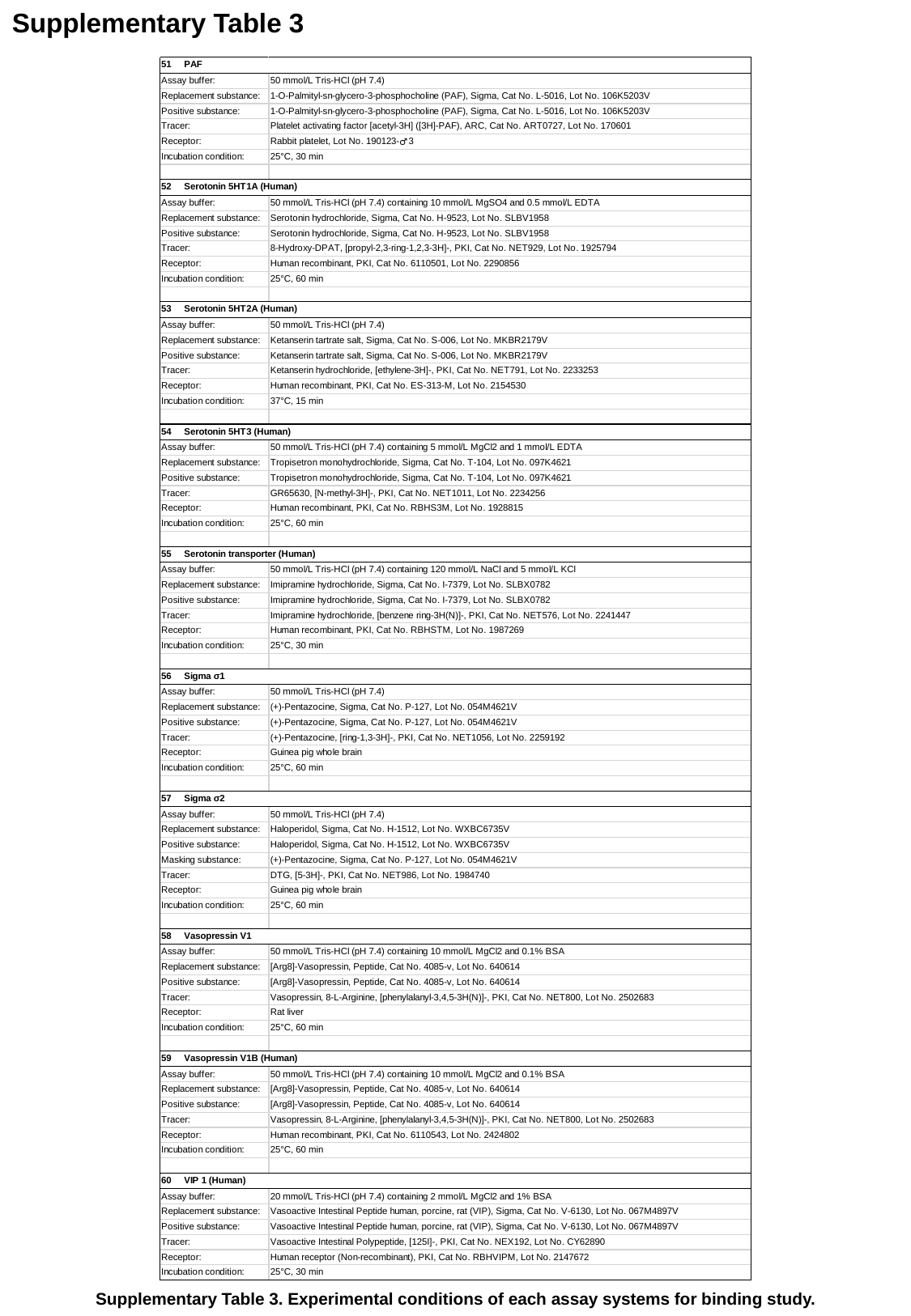

Supplementary Table 3
Supplementary Table 3. Experimental conditions of each assay systems for binding study.
