# Supplementary material for: STAT3 inhibition suppresses adaptive survival of ALK-rearranged lung cancer cells through transcriptional modulation of apoptosis
Source: NPJ Precis Oncol. 2022 Feb 28;6:11. doi: 10.1038/s41698-022-00254-y (PMC8885877; doi:10.1038/s41698-022-00254-y)
Supplement: Supplementary file 2 — REPORTING SUMMARY [file 41698_2022_254_MOESM2_ESM.pdf]

nature portfolio

Corresponding author(s): Shinji Takeuchi, Seiji Yano

Last updated by author(s): Dec 28, 2021

Reporting Summary

Nature Portfolio wishes to improve the reproducibility of the work that we publish. This form provides structure for consistency and transparency in reporting. For further information on Nature Portfolio policies, see our Editorial Policies and the Editorial Policy Checklist.

Statistics

For all statistical analyses, confirm that the following items are present in the figure legend, table legend, main text, or Methods section.

n/a	Confirmed	
☐	☒	The exact sample size (*n*) for each experimental group/condition, given as a discrete number and unit of measurement
☐	☒	A statement on whether measurements were taken from distinct samples or whether the same sample was measured repeatedly
☐	☒	The statistical test(s) used AND whether they are one- or two-sided<br>*Only common tests should be described solely by name; describe more complex techniques in the Methods section.*
☒	☐	A description of all covariates tested
☐	☒	A description of any assumptions or corrections, such as tests of normality and adjustment for multiple comparisons
☐	☒	A full description of the statistical parameters including central tendency (e.g. means) or other basic estimates (e.g. regression coefficient) AND variation (e.g. standard deviation) or associated estimates of uncertainty (e.g. confidence intervals)
☐	☒	For null hypothesis testing, the test statistic (e.g. *F*, *t*, *r*) with confidence intervals, effect sizes, degrees of freedom and *P* value noted<br>*Give P values as exact values whenever suitable.*
☒	☐	For Bayesian analysis, information on the choice of priors and Markov chain Monte Carlo settings
☒	☐	For hierarchical and complex designs, identification of the appropriate level for tests and full reporting of outcomes
☒	☐	Estimates of effect sizes (e.g. Cohen's *d*, Pearson's *r*), indicating how they were calculated

Our web collection on statistics for biologists contains articles on many of the points above.

Software and code

Policy information about availability of computer code

Data collection	Data were acquired with FUSION-SOLO Chemiluminescence Imaging System (Vilber Lourmat, Marne-la-Vallée, France), iMark™ Microplate Absorbance Reader (Bio-Rad, Hercules, CA, USA), Fluoroskan Ascent™ FL Microplate Fluorometer and Luminometer (Thermo Fisher Scientific, Waltham, MA, USA), FSX100™ fluorescent microscope (Olympus Corporation, Tokyo, Japan), and EnVision® Xcite Multilabel Reader (PerkinElmer, Waltham, MA, USA).
Data analysis	Data were analyzed with GraphPad Prism 8 (GraphPad Software, La Jolla, CA, USA).

For manuscripts utilizing custom algorithms or software that are central to the research but not yet described in published literature, software must be made available to editors and reviewers. We strongly encourage code deposition in a community repository (e.g. GitHub). See the Nature Portfolio guidelines for submitting code & software for further information.

Data

Policy information about availability of data

All manuscripts must include a data availability statement. This statement should provide the following information, where applicable:

- Accession codes, unique identifiers, or web links for publicly available datasets
- A description of any restrictions on data availability
- For clinical datasets or third party data, please ensure that the statement adheres to our policy

The datasets generated and/or analyzed during the current study are available from the corresponding author on reasonable request.

Field-specific reporting

Please select the one below that is the best fit for your research. If you are not sure, read the appropriate sections before making your selection.

☒ Life sciences ☐ Behavioural & social sciences ☐ Ecological, evolutionary & environmental sciences

For a reference copy of the document with all sections, see nature.com/documents/nr-reporting-summary-flat.pdf

Life sciences study design

All studies must disclose on these points even when the disclosure is negative.

Sample size	No sample-size calculation was performed, and generally accepted sample sizes were used, with reproducible differences between conditions indicating that the chosen sample sizes were sufficient.
Data exclusions	No data were excluded from the analysis.
Replication	For in vitro experiments, at least three independent biological replicates were performed with technical replicates per experiment whenever feasible. CRISPR KO screening and DNA microarray was performed in singlicate.
Randomization	For in vivo study, tumor-bearing mice were randomized based on tumor size before treatment to obtain an equal mean tumor size and similar distribution of individual sizes at the start of treatment.
Blinding	n/a

Reporting for specific materials, systems and methods

We require information from authors about some types of materials, experimental systems and methods used in many studies. Here, indicate whether each material, system or method listed is relevant to your study. If you are not sure if a list item applies to your research, read the appropriate section before selecting a response.

Materials & experimental systems

n/a	Involved in the study	
☐	☒	Antibodies
☐	☒	Eukaryotic cell lines
☒	☐	Palaeontology and archaeology
☐	☒	Animals and other organisms
☒	☐	Human research participants
☒	☐	Clinical data
☒	☐	Dual use research of concern

Methods

n/a	Involved in the study	
☒	☐	ChIP-seq
☒	☐	Flow cytometry
☒	☐	MRI-based neuroimaging

Antibodies

Antibodies used	Pimary antibodies for phospho-STAT3 (Tyr705, #9131), STAT3 (#4904), phospho-ALK (Tyr1604, #3341), ALK (#3633), phospho-ERK1/2 (Thr202/Tyr204, #4370), ERK1/2 (#4695), phospho-AKT (Ser473, #4060), AKT (#9272), BCL-XL (#2764), cleaved caspase-3 (#9664), cleaved PARP (#5625), FGFR1 (#9740), FGFR2 (#11835), FGFR3 (#4574), FGFR4 (#8562), MET (#8198), JAK1 (#3344), JAK2 (#3230), Tyk2 (#9312), gp130 (#3732), EGFR (#4267), IGF-1R (#9750), β-actin (#4970), and horseradish peroxidase-conjugated secondary antibody (#7074) were purchased from Cell Signaling Technology (Danvers, MA, USA).
Validation	All antibodies are commercially available and have been validated by the manufacturer.

Eukaryotic cell lines

Policy information about cell lines

Cell line source(s)	H3122 (EML4-ALK variant 1 E13; A20) cells were provided by Dr. Jeffrey A. Engelman (Novartis Institutes for BioMedical Research, Cambridge, MA, USA). H2228 (EML4-ALK variant 3a/b E6; A20) cells were purchased from American Type Culture Collection (ATCC, Manassas, VA, USA). The A925L (EML4-ALK variant 5a E2; A20) cell line was established from a surgical specimen obtained from a male Japanese patient (T2N2M0, stage IIIA) and provided by Drs. Fumihiro Tanaka and Hidetaka Uramoto (University of Occupational and Environmental Health, Fukuoka, Japan). Human lung embryonic fibroblast cell lines, MRC-5 and IMR-90 were obtained from RIKEN Cell Bank (Ibaraki, Japan).
Authentication	All cell lines were authenticated by short tandem repeat analysis at the National Institute of Biomedical Innovation (Osaka, Japan).

Mycoplasma contamination	All cell lines were tested negative for Mycoplasma infection at regular intervals using MycoAlertTM Mycoplasma Detection Kit (Lonza, Basel, Switzerland).
Commonly misidentified lines (See ICLAC register)	Commonly misidentified cell lines were not used.

Animals and other organisms

Policy information about studies involving animals; ARRIVE guidelines recommended for reporting animal research

Laboratory animals	5-6 weeks specific pathogen-free male SHO mice (Crlj:SHO-Prkdc scidHr hr) (Charles River Laboratories Japan, Inc., Kanagawa, Japan).
Wild animals	n/a
Field-collected samples	n/a
Ethics oversight	In vivo study protocol was approved by the Ethics Committee on the Use of Laboratory Animals and the Advanced Science Research Center, Kanazawa University (approval no. AP-173867).

Note that full information on the approval of the study protocol must also be provided in the manuscript.
